# A Systematic Review and Critical Analysis of the Role of Graphene-Based Nanomaterials in Cancer Theranostics

**DOI:** 10.3390/pharmaceutics10040282

**Published:** 2018-12-16

**Authors:** Teresa Viseu, Carla M. Lopes, Eduarda Fernandes, Maria Elisabete C.D. Real Oliveira, Marlene Lúcio

**Affiliations:** 1 CF-UM-UP—Centre of Physics of Universities of Minho and Porto, Departament of Physics of University of Minho, Escola de Ciências, Campus de Gualtar, 4710-057 Braga, Portugal; tviseu@fisica.uminho.pt (T.V.); eduardabfer@gmail.com (E.F.); beta@fisica.uminho.pt (M.E.C.D.R.O.); 2 FP-ENAS/CEBIMED—Fernando Pessoa Energy, Environment and Health Research Unit/Biomedical Research Centre, Faculty of Health Sciences, Fernando Pessoa University, 4249-004 Porto, Portugal; cmlopes@ufp.edu.pt

**Keywords:** systematic review, graphene-based materials, graphene oxide, reduced graphene oxide, nano-graphene, graphene quantum dots, theranostics, cancer

## Abstract

Many graphene-based materials (GBNs) applied to therapy and diagnostics (theranostics) in cancer have been developed. Most of them are hybrid combinations of graphene with other components (e.g., drugs or other bioactives, polymers, and nanoparticles) aiming toward a synergic theranostic effect. However, the role of graphene in each of these hybrids is sometimes not clear enough and the synergic graphene effect is not proven. The objective of this review is to elaborate on the role of GBNs in the studies evaluated and to compare the nanoformulations in terms of some of their characteristics, such as therapeutic outcomes and toxicity, which are essential features for their potential use as bionanosystems. A systematic review was carried out using the following databases: PubMed, Scopus, and ISI Web of Science (2013–2018). Additional studies were identified manually by consulting the references list of relevant reviews. Only English papers presenting at least one strategy for cancer therapy and one strategy for cancer diagnostics, and that clearly show the role of graphene in theranostics, were included. Data extraction and quality assessment was made by reviewer pairings. Fifty-five studies met the inclusion criteria, but they were too heterogeneous to combine in statistical meta-analysis. Critical analysis and discussion of the selected papers are presented.

## 1. Introduction

Despite all the efforts invested in therapeutic developments, cancer remains a leading cause of death worldwide, with a reported mortality of 8.8 million people in 2015. Moreover, the World Health Organization (WHO) and International Agency for Research on Cancer (IARC) predict a raise of all cancer cases to 21.2 million by 2030 [1,2]. Current therapeutic regimes for cancer treatment face important challenges, such as: (i) the use of high doses of actives to ensure their distribution at the target tissues, (ii) the need to combine several actives to increase therapeutic efficiency and reduce multidrug resistance mechanisms (MDR), and (iii) damage of healthy tissues and severe toxicity because of (i) and (ii). 

**Figure 1:**
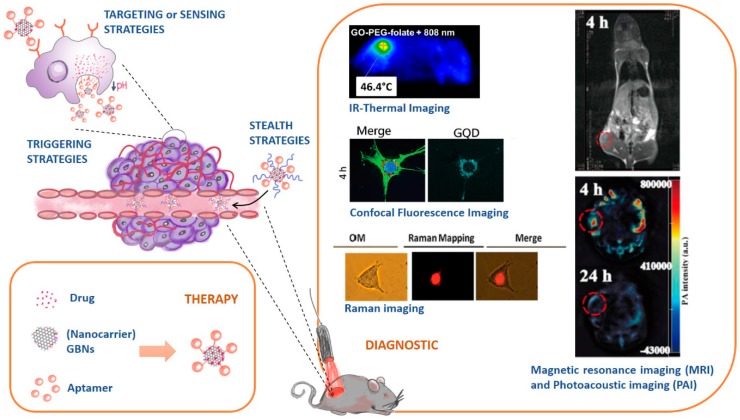
Targeting, triggering, and stealth strategies in cancer therapy conjugated with diagnostics (theranostics). Diagnostic images were adapted from References [5,6,7,8] with permission from Elsevier and John Wiley and Sons.

Nanomedicine is potentially one of the best strategies to deal with such challenges, as it provides breakthrough improvements over classical therapies. Indeed, nanocarrier systems possess extremely large surface areas convenient for loading multiple therapeutic actives and/or other elements with stealth, targeting, triggering, or imaging roles [3,4]. Additionally, the concept of a nanocarrier holds the promise of taking the actives to the target tissue, thereby reducing the exposure of healthy tissues to toxic effects. This assumption is further reinforced in cancer, as a preferential accumulation of nanocarriers in tumor sites has been observed due to the enhanced permeability and retention (EPR) effect [3,4]. Hence, nanocarriers for cancer therapy can be thoroughly tuned to integrate multiple strategies in one system (Figure 1):

- Stealth strategies: nanocarriers can be coated with polymers (e.g. Polyethylene glycol, PEG) to keep them invisible to the immunity system and increase their circulation time;
- Targeting or sensing strategies: nanocarriers can be functionalized with ligands that are recognized by receptors overexpressed in cancer cell tissues;
- Triggering strategies: nanocarriers’ composition can be sensible to stimulus (e.g. pH, temperature changes) and releasing their cargo accordingly.

The multipurpose character of the nanocarrier systems has been further explored in the context of developing theranostic (therapeutic + diagnostic) tools for fighting cancer that combine several therapies with imaging strategies to monitor distribution of the therapeutic agents in the body (Figure 1). Currently, multiple combinations of different therapeutic and diagnostic strategies are being applied to achieve a theranostic effect. As each strategy has inherent advantages and limitations, the combination of complementary strategies can result in a synergic theranostic effect. Tables S1, S2 and S3 (Supplementary material) summarize some of the most important therapeutic strategies (drug and gene therapy, phototherapy (PTT), magnetic hyperthermia (MHT), and photodynamic therapy (PDT)), and diagnostic strategies (therapy guiding by fluorescence imaging (FI), two photon fluorescence imaging (2PFI), infrared thermal imaging (IR-TI), Raman imaging, X-ray imaging, magnetic resonance imaging (MRI), positron emission tomography (PET), single-photon emission computed tomography (SPECT), computed tomography (CT), photoacoustic imaging (PAI), and ultrasound imaging (USI)) that are being used in nanotheranostic systems [9,10,11,12,13,14,15,16,17,18,19,20,21,22,23,24].

After its ground breaking and surprising discovery in 2004 [25], graphene—a two-dimensional (2-D) nanomaterial made of single-layered carbon atoms packed in a honeycomb lattice—has been widely explored for a great number of applications including quantum physics, nanoelectronics, energy storage, and catalysis [26,27]. Since the first publication on the use of graphene as a nanocarrier system for anticancer drugs delivery in 2008 [28], graphene and graphene-based nanomaterials (GBNs) have also captivated the enthusiasm of researchers for its promising biomedical applications, translatable in an increasing number of publications [29]. 

**Figure 2:**
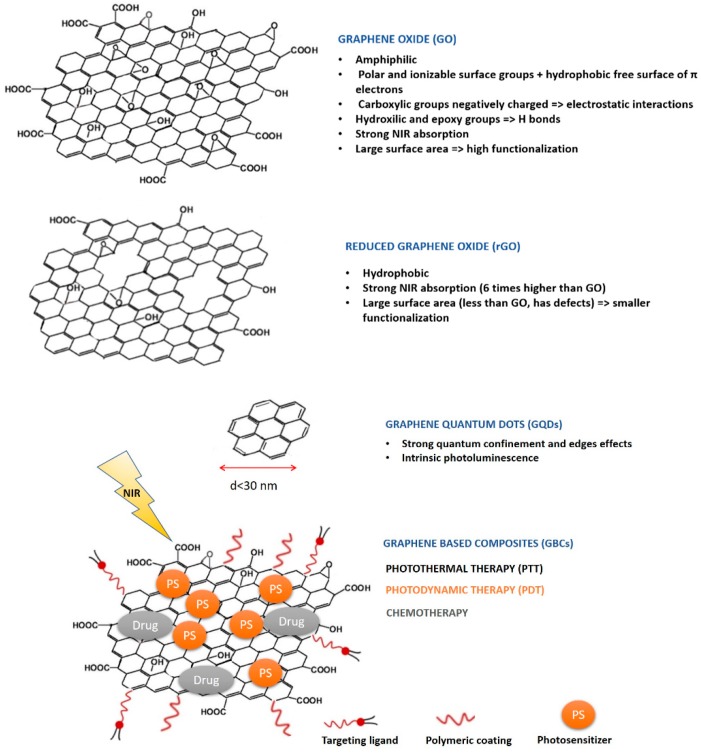
Schematic illustration of different graphene-based nanomaterials (GBNs) and graphene-based composites functionalized with targeting ligands, polymeric coating photosensitizer (For PDT therapy), drug (for chemotherapy). Near infra-red (NIR) light absorbed by these nanomaterials is required for PTT, PDT, and to possibly trigger drug release.

GBNs include graphene derivatives, such as graphene oxide (GO), nanographene oxide (NGO), reduced graphene oxide (rGO), and graphene quantum dots (GQDs), which are also designated as nano-reduced graphene oxide (nano-rGO) [30,31]. The appealing optical and physicochemical properties of GBNs are well recognized and have been explored in the development of theranostic nanosystems for the treatment and diagnostic of cancer [31,32,33]. In this context, researchers take advantage of the inherent properties of GBNs (e.g., fluorescence emission, NIR light absorption, photothermal properties, and typical Raman bands) to develop therapeutic (PTT) and diagnostic (FI, 2PFI, IR-TI, and Raman imaging) approaches [13,16,34]. Alternatively, researchers take advantage of GBNs’ easy functionalization (owed to the large surface-volume ratio and sp^2^ network that allows π–π stacking interactions) to add other components that are aimed to enhance or complement GBNs properties and to achieve synergic theranostic effects [30,32]. These hybrid nanostructures encompass graphene-based composites (GBCs) that result from GBNs functionalization with nanoparticles, polymers, imaging probes, radionuclides, drugs, nucleic acids (or other bioactives), photothermal (PT), and photosensitive (PS) compounds [30,32]. Figure 2 illustrates the different types of GBNs and provides an example of GBC, as well as some of GBNs most important properties and their main applications in cancer theranostics. 

Despite the multitude of GBNs developed for theranostic purposes, and the increasing amount of published research articles and reviews on GBNs, it is important to address the question of the actual function of GO in the systems developed, as well as to evaluate the synergic value of the GBCs. Indeed, it is not always clear if the functionalized GBNs hybrid systems result in more effective theranostic strategies than the isolated components, as many studies fail to use appropriate controls to study the separate elements. Therefore, this lack of clarity drives the need for this systematic review. Accordingly, the objectives of our study are to systematically review the literature for qualitative and/or quantitative evidence of synergic effects of GBCs in cancer theranostics, as this is a determinant to understand the role of GO in the total system developed and to further develop other systems with improved characteristics. Our secondary aim is to provide an overview of the therapeutic anticancer efficiency of the nanosystems developed in vitro and/or in vivo. Moreover, the concerns raised with toxicity and biocompatibility of neat GO are still under extensive debate [35,36,37]. The toxicity of GO has been associated with several parameters, among which are included size and topological defects of GO flakes and their propensity to aggregate [38,39]. Bigger sizes, higher defects, and smaller colloidal stability may contribute to reactive oxygen species (ROS) generation and consequent cellular toxicity [38,39]. Furthermore, this type of reactivity is not desirable when, for instance, the objective is conjugating the GO material with metal-based nanoparticles or metal-based drugs [38]. These concerns invoked a critical analysis of the reported studies taking into consideration the characterization of GO in terms of size and colloidal stability and the toxicity evaluation performed in the selected studies. To the best of our knowledge there are no similar reported reviews emphasizing the role of GBNs in cancer theranostic and this is the first systematic effort to provide an in-depth critical analysis of the updated literature to serve as a guide for researchers working in this field.

## 2. Methods

This review followed PRISMA guidelines [40] and the correspondent checklist is presented in the supplementary material (Table S4)

### 2.1. Eligibility Criteria

In this review we analyzed studies proposing the development of GBNs (GO, NGO, rGO, GQDs) or GBCs for theranostic use in cancer.

Studies were included according to the following criteria: (1) studies were published in English with full text available; (2) cancer was the target disease; (3) at least one type of GBNs was present in the proposed nanosystem; (4) studies presented at least one strategy for therapy and one strategy for diagnostics, and GBNs had a role in at least one of these strategies; and (5) studies were evaluated in vitro using cellular lines and/or in vivo using animal models. Only research articles were considered, reviews, commentaries, letters, or editorials were excluded. The references in reviews were, however, manually consulted. 

Of interest are interventions in studies addressing the role of GBNs in the overall therapeutic effect or diagnostic performance and the synergic effect obtained by GBNs conjugated with other components. In addition, we considered interventions that evaluate GBNs toxicity as this is also a relevant factor to obtain an efficient theranostic system. Only studies that evaluate GBNs as a stand-alone component of the hybrid GBCs and compare the efficiency of the hybrid GBCs with the isolated components were included.

Primary outcomes were the ones related with theranostic effects: types of strategies used in therapy and diagnostic and measures of therapeutic efficiency. Secondary outcomes were related with the characterization of the GBNs developed and with the toxicity of the systems.

### 2.2. Information Sources, Search Strategy, Study Selection, and Data Collection Process

The literature search strategy was conducted in electronic databases (PubMed, Scopus, and ISI Web of Science), which was complemented by hand searching in the reference lists of included studies or relevant reviews identified through the search. The search was based on a pre-determined series of keywords related with graphene-based materials and their application in theranostics. The following keywords were used in PubMed: (((graphene[Title/Abstract]) OR graphene oxide[Title/Abstract]) OR graphene quantum dots[Title/Abstract]) AND theranostic[Title/Abstract] and these were adapted to the syntax and subject headings of the other databases. No study design or language limits were imposed on the search. However, the time of publication was limited to the last 6 years (2013–2018). The last search was run on 15 October 2018. 

To facilitate collaboration among reviewers during the study selection process, literature search results were uploaded to Endnote X7^®^ software and included in a OneDrive shared folder. Endnote X7^®^ allowed an automatic exclusion of duplicates from the three databases searched, and a manual search of duplicates was processed afterwards.

A two-stage process was used during the study selection. In the first stage, the review authors screened the title, abstract, and keywords against the eligibility criteria. Whenever there was uncertainty about the eligibility of the publication, the study was not excluded. From this stage full text of all the eligible and uncertain studies were included to be analyzed in the second stage. In stage two, review author pairs have screened the full text publications and decided whether these met the eligibility criteria. Neither of the review authors were blind to journal titles or to authors and institutions of the studies analyzed. For data collection from the selected studies, we developed a data extraction sheet, which was pilot tested and optimized accordingly. One review author extracted the data and the second reviewer author confirmed the extracted data. Any disagreement between reviewers was resolved after discussing and reaching a consensus.

### 2.3. Data Items and Quality Assessment

The information extracted from each study included: (1) characteristics of the GBNs developed in each study, including composition, components ratio, size, surface charge, colloidal stability analysis, and characterization techniques used; (2) in vitro and/or in vivo models used, detailing the concentration and doses of the complete system versus the separate components and controls, as well as the irradiation procedures (potency) power, wavelength, and duration; (3) theranostic outcome described in terms of diagnostic/therapeutic strategies used and therapeutic efficiency achieved (measured by hyperthermia effect, cellular viability and tumor volume reduction or evaluated by histological observations); (4) toxicity outcome (measured by cellular viability, animal weight variations, and animal survival rate, or evaluated using histological observations).

Following the full-text selection, the studies were assessed for methodological quality via pairs of reviewers working independently using criteria from QualSyst tool for quantitative/qualitative studies [41] adapted to meet the specific needs of this systematic review (Table S5 Supplementary material). All the studies selected met the minimum threshold for inclusion.

## 3. Results

A total of 55 studies were identified for inclusion in the current review. The search conducted in the databases retrieved a total of 659 citations (95 from PubMed, 184 from Scopus, and 380 from ISI Web of Science). A manual selection included 84 references found in updated and extensive reviews that contained studies on the use of GBNs for cancer theranostics [16,30,42,43]. After duplicate elimination, 490 studies remained. 

Of these, 326 studies were rejected after title, abstract, and key words reviewing for not comprising the eligibility criteria. These rejected articles included: 1 study that was not published in English, 109 reviews, and 6 book chapters. The option to exclude reviews and book chapters was to analyze studies in their original published form, avoiding introducing bias from analyzing the studies after they have been analyzed by other authors. Three editorials and three proceedings/short communications were also excluded because the information contained in these publications was too scarce regarding the outcomes we wished to analyze. Another eligibility criterion was to include studies that developed nanosystems that possessed at least one type of GBN in their composition and the application of this criterion has rendered the exclusion of 189 studies. Finally, in the first screening phase, we also excluded studies for which cancer was not the target disease.

The full text of the remaining 164 studies was carefully examined. From this analysis, 109 studies were rejected for not fitting in the inclusion criteria. The first criterion defined that studies should possess at least one strategy for therapy and one strategy for diagnostics, otherwise we could not consider the nanosystem developed as adequate for theranostic applications. Many studies failed to accomplish this criterion, and the authors often designated their developed nanosystem as a theranostic system, only because they envisaged the possibility of such a system to be used in both therapy and diagnostics, but they do not present evidence for both applications. In other cases, authors claimed that their GBNs were developed for diagnostic purposes, but they had to label them with extrinsic probes to be able to guide its cellular uptake, which dismisses the purpose of using GBNs or GBCs because of their intrinsic diagnostic properties. The application of this criterion led to the exclusion of 65 studies. The second criterion defined that GBNs must have a role in the nanosystem developed contributing either to diagnostics or therapy. This role can be defined as synergic with other components in the system such that in the final system the graphene/components individual intrinsic properties are enhanced. Several authors classified the final effects of their nanosystems as resultant from a synergic contribution of GBNs and additives, but their study misses evidence of the individual capacities of GBNs or additives. This second criterion led to the exclusion of 41 studies. Finally, the third criterion determined the inclusion of studies a bit more advanced than fundamental research and excluded studies in which the developed nanosystems were neither tested in vitro nor in vivo.

**Figure 3:**
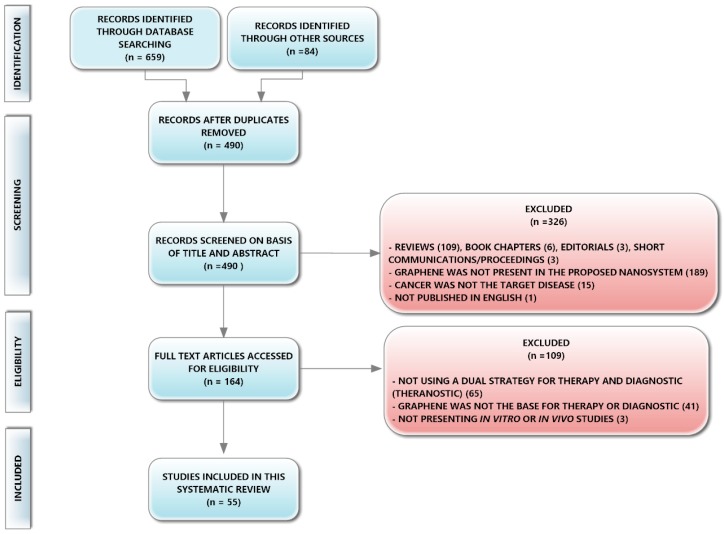
Flowchart diagram of the study selection for this systematic review.

From this screening and eligibility process 55 studies met the abovementioned inclusion criteria, being eligible to be included in this review (see flow chart diagram in Figure 3).

All 55 studies report the development of GBNs alone [44,45] or often functionalized with different additives [5,6,7,8,46,47,48,49,50,51,52,53,54,55,56,57,58,59,60,61,62,63,64,65,66,67,68,69,70,71,72,73,74,75,76,77,78,79,80,81,82,83,84,85,86,87,88,89,90,91,92,93,94] for cancer theranostic applications. Each of these studies presented the assessment of the individual role of GBNs either in therapeutic or diagnostic applications. Within the complete set of studies, the therapeutic performance was evaluated in cellular [6,49,50,52,53,55,63,68,70,80,81,82,83,84,85,86,89,94], or animal models [46,71,77], or both [5,7,46,47,48,51,56,57,58,59,60,61,62,64,66,69,72,73,74,75,76,78,79,81,87,88,90,91,92,93]. In the case of cellular studies, GBNs were incubated at a defined concentration or in a range of concentrations and some studies involved the application of GBNs solely [46,53,54,55,63,68,71,78,82,91] or conjugated with the exposure to a laser source of a defined potency, wavelength, and time [5,6,7,8,44,45,47,48,49,50,51,52,56,57,58,59,60,61,62,64,65,66,67,69,70,72,73,74,75,76,77,79,80,81,83,84,85,86,87,88,89,90,92,93,94]. With the exception of few studies [45,47,87,94], the laser used is a near infrared light source (NIR) that has a trigger effect for drug or active release [5,50,51,62,79] or is the promotor of PTT effects [5,6,46,50,51,58,59,60,61,62,64,66,69,70,72,73,74,75,76,77,79,86,90,92,93] or PTT+PDT effects [7,48,49,52,56,57,67,81,83,85,88]. Cell viability was assessed by state-of-the-art methods, such as MTT [5,6,7,44,45,47,48,49,50,53,54,56,57,58,59,60,62,64,66,68,70,73,74,80,82,83,84,85,86,88,90,92,94], CCK-8 [51,52,63,65,67,69,72,79,87,89,91,93], SRB [75,76,78], trypan blue [61,82,83], alamar blue [8], or calcein AM [81]. In vivo studies used xenografted animals with cancer tumor cells, and the GBNs were injected intravenously alone [54,71,78,81,91], or in conjugation with laser exposition [5,7,8,45,46,47,48,51,56,57,58,59,60,61,62,64,65,66,67,69,72,73,74,75,76,77,79,87,88,89,90,92,93,94], after which, therapeutic efficiency was evaluated. Therapeutic effect was measured in terms of: tumor volume, survival rate, and tumor tissue damage evaluated histologically. In vivo toxicity was assessed via the effects in body weight or by histological damage of a non-therapeutic form of the nanosystem (e.g., without laser exposition). In vitro toxicity assessment is often done in the same cancer cell line used for therapeutic evaluation; however, some studies use a cell line representative of healthy tissues for this assessment [5,47,48,49,53,57,58,61,62,67,68,70,81,82,85]. The in vitro toxicity assessment is also called “dark cell viability” as it is always performed in the absence of laser light exposition to infer the cytotoxicity of a non-therapeutic form of the nanosystem.

Because physical–chemical factors greatly impact the resultant toxicity and biocompatibility of graphene, characterization features, such as evaluation of size (lateral dimension and thickness), charge, surface coating, and colloidal stability, should be also considered as an outcome of the studies [38,39]. However, in this regard, the great majority of the studies failed to provide complete information. From the 55 studies, only 3 studies have presented these parameters [56,87,91].

The summary of study characteristics for which data were extracted are presented in Table 1 (type of GBNs, characterization features, and GBNs’ role in therapy or diagnostic, including the reference of figures from each paper where this role is demonstrated), Table 2 (types of therapeutic strategies, trigger strategies, and diagnostic strategies), and Table 3 (cellular and animal models, therapeutic and toxic outcomes in vitro and in vivo, doses of GBNs and actives administrated, actives’ loading efficiency and laser characteristics). 

From the observation of Table 1 it is possible to conclude that most studies used GO (17 studies), followed by the other types of GBNs: rGO (13 studies), GQDs (13 studies), and NGO (12 studies). This preference must relate to the enriched oxygen surface of GO (carboxylic, epoxy and hydroxyl groups) that favor a broad extent of interactions and functionalization opportunities [30,31].

**Figure 4:**
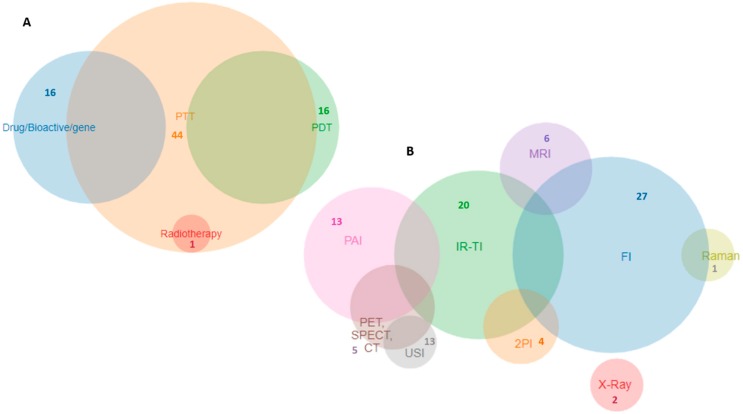
Venn chart of therapeutic (**A**) and diagnostic (**B**) strategies presented in this systematic review.

From the observation of Table 2 it is possible to conclude that the most explored therapy modality was PTT (44 studies), followed by PDT (16 studies), and chemotherapy (16 studies). The most explored synergic effect was the combination of PTT+PDT (14 studies), followed by chemotherapy+PTT (8 studies). Doxorubicin (DOX) was the anticancer drug used in most chemotherapy modalities (16 studies) and the drug release was often adjuvated by a trigger effect (10 studies), such as NIR and pH changes. The most explored diagnostic modality was FI (27 studies), followed by IRTI (20 studies) and PAI (13 studies). All studies presented the combination of at least one therapy strategy in combination with at least one diagnostic strategy, and the most complex system [48] combined two therapeutic strategies (PTT+PDT) with four diagnostic strategies (2PFI+MRI+CT+PAI). Figure 4 presents a Venn chart with the relative proportion of therapy and diagnostic strategies explored in the 55 studies analyzed. 

## 4. Discussion

Because the study designs, interventions, and reported outcomes varied noticeably, we focused on the description of the studies for which we systematically organized their results in Table 1, Table 2 and Table 3. We will discuss their relevance, and their limitations, in a qualitative critical analysis instead of a meta-analysis. 

The aim of this systematic review was to critically analyze the theranostic role of GBNs when it is the only component of the nanosystem or is instead incorporated in more complex nanosystems. First, the notion of theranostics presupposes the existence of a combined therapeutic and diagnostic strategy in the same system. However, not so uncommonly, studies present their developed nanosystems as a theranostic tool, even when they lack experimental evidence for both strategies. Though these studies may develop promising systems for theranostic applications, they should not be considered as such, only based in a theoretical assumption. Many of these studies have been introduced in previous reviews as theranostic nanosystems, but we have excluded them from the current review, as the inclusion criterion defined that only experimentally proved theranostic systems should be considered

The reason for the systematic scrutiny also came from the perception that the GBNs’ component of the system is not always analyzed for its individual effects either in therapy or in diagnostic, and without this analysis, its role in the entire system is compromised. It also compromised the assumption of a synergic effect of the entire system when the individual effects of each individual component are not evaluated. 

Considering these aims, we will propose a discussion based on the role of GBNs in therapy, diagnostics, and toxicity of the overall theranostic systems, and we will also comment on the perspectives of a future clinical translation.

### 4.1. Role of Graphene-Based Nanomaterials in Therapy

#### 4.1.1. Overview of the Different Therapeutic Strategies

GBNs intrinsic properties have converted these nanomaterials into a very attractive choice as drug and gene delivery systems. The combination of a 2-D structure with a large surface area and the existence of delocalized π electrons, as well as chemical polar groups that exist mainly on the GO surface, grants high drug loading ratios of lipophilic and hydrophilic actives [32]. Indeed, within the chemical surface moieties of GO, the epoxy and hydroxyl groups can establish hydrogen bonds with actives, while the carboxylic acid group offers negative surface charge for electrostatic interactions with positively charged molecules (Figure 2). Furthermore, actives can establish hydrophobic and π-π stacking interactions with GBNs [32]. Finally, active loading can be achieved via covalent and noncovalent surface functionalization of GBNs. Such a diverse range of possible interactions has given GBNs an important role in drug delivery, as it has been documented that drug loading ratios can achieve 200 wt%, which is exceptionally high compared to any other drug nanocarrier system [95].

Among the studies included in this review, DOX is the most used drug [5,8,50,53,54,62,63,68,74,75,76,79,89], as it can be efficiently loaded in GBNs via a simple π-π stacking interaction, or alternatively, it can be bonded to GBNs via ester linkages [62,76]. The drug loading achieved with the GBN systems can in fact be higher than that achieved with other nanocarrier systems. For instance, the commercial liposomal formulations of DOX, Caelyx^®^, and Doxil^®^ have a drug loading of 16 wt%, while most GBNs formulations of this review (Table 3) are able to reach higher drug loading values, from 55 wt% to 133 wt% [5,50,54,71,75,76,79]. However, the drug loading capacity is not the only appealing feature of loading DOX in GBNs. The possibility of exploring triggering strategies that can release the drug under different stimuli is also attractive, as the idea is to carry the drug throughout the body with minimal release in the healthy tissues, and a higher release in the cancer target tissues. In the cases that DOX has been bonded to GBNs via ester linkage, this linkage can be broken by physiological esterases that are often overexpressed in cancer tissues or the drug release can be triggered externally using NIR radiation [62,76]. NIR radiation is a triggering strategy commonly used to release DOX from GBNs [5,8,62,76,79]. Moreover, DOX is positively charged at physiological pH, while the carboxylic groups of GBNs are negatively charged, which favors an electrostatic interaction. This was used as a triggering strategy for a pH-dependent release of DOX in several studies in the current review [5,53,63,71,79,80]. As in the tumor microenvironment, intracellular lysosomes and endosomes are acidic, the carboxylic groups of graphene become non-ionized and the electrostatic interaction between drug and GBNs ceases. Additionally, one study has used an alternative triggering strategy by functionalizing GBNs with a pH labile linker that, in the face of the acidic tumor environment, releases the drug [8]. Conjugation of GBNs with iron oxide nanoparticles confers superparamagnetic properties to the overall nanosystem, and this was another strategy explored for a pH-assisted magnetic triggering of DOX [50]. 

Besides the use of GBNs as carriers of DOX, one study has used GBNs loaded with paclitaxel (PTX), also with a very successful drug loading (90 wt%) [91] in comparison with the commercial PTX formulations Taxol^®^ (1 wt%) and Abraxane^®^ (11 wt%). In this study, PTX was non-covalently immobilized on the NGO surface via a π-π stacking interaction and hydrogen bonding, and PTX release was also triggered using pH. At acidic pH (5.0), characteristic of tumor tissues and intracellular lysosomes, PTX was released in 30% greater quantities than at blood and healthy tissues pH (7.4) [91]. However, the authors of this study do not present an explanation for such pH-triggered drug release, and this is not as evident as in the case of DOX, since PTX is neutral at all the pH values tested and NGO surface groups will be more protonated at acidic pH, favoring the hydrogen bonding with PTX.

Other than drugs, bioactives have also been used with a chemotherapy purpose. Natural compounds with anticancer effects have hampered pharmaceutical uses due to their small bioavailability and poor stability [96]. Nanocarriers have given these compounds new prospects for clinical application by improving bioavailability and protecting them from early metabolization or degradation [96]. GBNs offer the additional advantage of providing higher payloads than the conventional nanocarriers, and in this review, GBNs were used to load curcumin (Cur) [78], resveratrol (Res) [58], and berberine hydrochloride (BHC) [82] with loadings of ≈ 41 wt%, 70 wt%, and 88 wt%, respectively. In contrast to Cur and Res that were loaded in GBNs by π-π stacking interactions, BHC was functionalized on the surface of GQDs by means of a biocompatible linker (Cys-HCl). At acidic pH characteristic of tumor cell tissues, there was an increased release of the bioactive resulting from breaking of the bonds between BHC and GBNs, therefore pH was also used in this case as a triggering mechanism.

In an interesting approach to chemotherapy, Yu and coworkers have used the peptide H_2_N-Gly-Ala- Gly-4Hyp-Pro-Tyr-CONH_2_ (AAP10), which is a bioactive that has its mode of action based on a bystander effect [90]. The bystander effect refers to the ability of injured cells in transmitting cytotoxic signals to the nearby cells, which become more sensitive to chemotherapy and PTT damages. This effect is primarily controlled by a connexin-mediated gap-junction intercellular communication (GJIC). Sadly, most cancer cells possess deficient GJIC, and in addition, tumors are 3-D structures, which can restrain the efficacy of the bystander effect. Consequently, the inexistence of a bystander effect impairs the propagation of damage between cells and some cells become shaded to therapy. Thus, both chemotherapy and PTT fail to kill all tumor cells, which results in cancer recurrence. Therefore, enhancing the bystander effect in tumor cells is an encouraging strategy to increase PTT and chemotherapy efficacy. In this regard, AAP10 was loaded in rGO to elevate the protein activity of connexins, thereby promoting the transfer of toxic signals or toxics between adjacent cells in tumors via a bystander effect [90].

The large surface of GBNs is also attractive for the development of a multipotential nanosystems that comprise, not only the drug, but also other components to enhance therapeutic performance and reduce toxicity. This is the case of targeting or sensing ligands that are used to specifically bind to overexpressed receptors existent in cancer cells, reducing internalization by normal cells, thereby reducing off-target toxicity. The overall targeting strategies used in the studies reviewed are summarized in Table 4:

The same favorable properties of GBNs referred to above for drug and bioactive delivery applications can be extended to gene delivery applications. Indeed, even in the absence of cationic groups, GBNs have demonstrated the capacity to effectively condense genetic material through π-π stacking interactions, protecting the nucleic acids from endonucleases degradation. Many studies have emphasized the role of GBNs in gene delivery in vitro [30]; however, there is a need to establish their potential in vivo, paying special attention to its safety profile, transfection efficiency, and biodistribution. The only two studies included in this systematic review that are aimed at genetic therapy, although promising in vitro, also lack in vivo validation [84,94]. 

Besides chemotherapy, and contrastingly to a previous review that covered studies reported from 2012–2014 [97], we found that the most explored therapeutic strategy of GBNs is PTT [5,6,46,50,51,58,59,60,61,62,64,66,69,70,72,73,74,75,76,77,79,86,90,92,93]. In the time frame of that review, chemotherapy was the focus of using GBNs, while in the last 6 years, covered by this systematic review, researchers have shown the appealing role of GBNs in PTT. 

By being 2-D materials, GBNs have a broad absorption in the NIR region (700–1100 nm) due to their narrow band gap that expands the light absorption to NIR region. Upon NIR radiation absorption, GBNs can convert it into thermal energy, which causes a temperature rise and cellular damage or death via hyperthermia (please see Table S1 of supplementary materials for a more detailed explanation of the PTT mechanism). Simultaneously, this process of temperature rise reduces GO and releases gases, creating a microcavitation environment from gas bubbles formation and collapse. The microcavitation environment, if created in a cellular media, is in turn responsible for cell death [98]. Most GBNs have been reported to have a good photothermal conversion efficiency that enables reaching a high PTT efficiency with a low power density of NIR light, and this potentiality was used in two studies herein reviewed, where PTT efficiency was exclusively obtained from GQDs NIR absorption capacity [44,45]. In other studies, the PTT efficiency of GBNs was further enhanced via conjugation with other narrow bandgap materials due to their increased NIR absorption, such as: Au based nanomaterials [6,61,66,68,70,79,87], Ag based nanomaterials [76]; cyanine and cysteamine based dyes (e.g., Cy5.5, Cy7, CysCOOH) [56,72,73,85]; IR780 iodide dye [65]; indocyanine green dye [59,69,86,93]; and Bi_2_Se_3_ nanoparticles. Another strategy to increase cell death by hyperthermia is to combine magnetic hyperthermia with PTT efficiency via the conjugation of GBNs with magnetic nanoparticles made of transition metals, such as: Fe, Ni, Co, Mn and its oxides (e.g., MnWO_4_, and IO-supermagnetic iron oxide nanoparticles) [5,60]. In comparison with GO, rGO has 6 times higher NIR absorption, and as such, rGO are more efficient GBNs for PTT applications (Figure 2). Therefore, another strategy used in these studies was coating GBNs with polydopamine (pDA), as it reduces GO to rGO, thereby increasing NIR absorption [59,90].

Although the studies selected in this systematic review demonstrate the important role of GBNs in the PTT therapeutic strategy, some limitations can be also pointed out. First, the photothermal conversion efficiency should be calculated and reported, since a high demonstrable photothermal conversion efficiency avoids the use of high power NIR laser density that can cause serious burn wounds and tissue shrinking [99]. In fact, some authors mentioned that upon NIR laser irradiation, a noticeable black round mark persisted on mice skin, which is direct evidence of the generation of excessive local heating [61]. Accordingly, there is a big discrepancy of power used from study to study, varying from small values of 6.5 mW/cm^2^ [44] to high values of 6.67 W/cm^2^ [61], and without the value of photothermal conversion efficiency, it is very difficult to analyze which GBNs or GBCs are more effective. Second, photostability of the overall GBCs should also be demonstrated.

Besides PTT, PAT is another therapeutic strategy in which photon energy is converted in an acoustic shockwave (PA wave) to kill cancer cells, being different from PTT where photon energy is converted to heat to damage or kill cancer cells [72]. Therefore, in PTT, there is an application of a continuous laser radiation, whereas in PAT the duration of the laser pulse is shorter than the thermal diffusion time [72]. This strategy was used by Qin and co-workers to conjugate a dye with GO, and the fluorescence quenching of this dye promoted by GO reduced the release of the absorbed energy via a radiative transition while increasing the non-radiative transition [72].

Recently, GBNs have also been used in PDT for its capacity of forming complexes with photosensitizers (PS) via π-π stacking and hydrophobic interactions. The different PS used in the studies reviewed can be identified in Table 2. The energy transfer from the laser NIR light source to PS will cause reactive oxygen species generation (ROS), which are ultimately responsible for cell damage and death (please see Table S1 of supplementary materials for a more detailed explanation of the PDT mechanism). This strategy was used in several studies of this review that generally took advantage of the synergistic effect of the combination of two therapy modalities: PTT + PDT [7,45,47,48,49,52,56,57,67,81,83,85,87,88]. These strategies can be a good alternative to drug delivery as they do not require the cellular internalization of GBNs. Given the wide ranges of lateral size dimensions and thickness of the materials reported herein (Table 1), it would be difficult for them all to succeed in a context of drug/gene delivery, where a control of sizes <100 nm is required to enter the cell and <40 nm to access the nucleus. However, the restricted depth reached by UV light debilitates the PDT effect. Accordingly, some studies have used the strategy of combining GBNs with UCNPs, which can convert the high depth penetration NIR light into high-energy photons (UV/vis) [48,57,85].

#### 4.1.2. Critical Comparison of Therapeutic Outcomes

By analyzing Table 3, it is possible to conclude that all the studies reported were successful in killing cancer cells in vitro; however, when evaluating therapeutic outcomes in vivo, it is possible to find bigger differences in the sense that some formulations show much higher anti-tumor effects by ablating the tumor completely [5,7,44,46,51,56,58,59,64,65,66,67,69,73,77,79,87,88,90,91,92], while other formulations are not able to achieve a complete ablation of the tumor [45,47,48,54,57,60,61,62,71,72,75,76,78,93].

One immediate analysis is that GBNs made of rGO were more successful in achieving a tumor elimination (9 studies out of 10 were able to ablate tumor between 3 to 16 days), most probably because in all studies, PTT was the therapeutic strategy alone or in combination with other strategies and rGO presents a high NIR absorbance (6 times higher than GO or NGO), thereby possessing intrinsic higher photothermal conversion to kill cells via hyperthermia. Besides the PTT efficiency, the high tumor uptake might be the explanation for the success of these formulations in tumor ablation. Table S6 of the supplementary materials summarizes the biodistribution information of some of these formulations than can be correlated to the successful therapeutic outcomes obtained. In three other studies with rGO nanomaterials that were able to eradicate the tumor, there is no biodistribution information available to understand tumor uptake and accumulation [59,66,90]. However, some characteristics of these formulations might explain their success in tumor ablation. rGO-GSPs formulation was able to ablate tumors in 2 days with a survival rate ≥40 days using PTT as therapy strategy [66]. The reason for such a successful therapeutic outcome might be related to the synergic effect obtained by conjugating rGO (already a high thermal conversion nanomaterial) with gold superparticles (GSPs), which endowed the rGO-GSPs formulation with NIR absorption and enhanced photothermal conversion properties due to the strong plasmonic coupling effect [66]. Indeed, after administration of this formulation, the surface temperature of the tumor increased rapidly from ≈30 °C to ≈58 °C within 5 min of 808 nm laser irradiation at a power density of 0.8 W/cm^2^ [66]. AAP10-pDA/rGO formulation was also very effective at promoting tumor ablation in 7 days [90]. In this case the success of the formulation might be related with the bystander effect of the AAP10 peptide that, as already explained, allows for damage effects resulting from PTT to be effectively transferred from one cell to the adjacent cells [90]. Finally, two formulations containing both rGO and ICG were developed for a PTT strategy, but the therapeutic outcome obtained was quite different. ICG-PDA-rGO formulation was able to ablate tumors in 3 days with a survival rate ≥40 days [59], while rGO-mfHSA was only able to reduce tumor growth by 4-fold in 20 days [93]. Although biodistribution data is not available for comparison purposes, and although both formulations contain two synergic components for PTT (ICG + rGO), the most effective formulation contains pDA that has been described as able to increase NIR absorption, thereby being a third additional synergic component for PTT [59]. 

In GBNs formulations made of GO or NGO loaded with DOX for a chemotherapy strategy used as only strategy [71] or combined with PTT [62,75,76], the therapeutic outcomes are improved regarding the use of free DOX, but the tumors are not totally ablated. The only exception is observed for the formulation GO/MNWO_4_/PEG that was able to eliminate tumor in 12 days (and no remission observed until the end of the study after 16 days) [5]. In this formulation there is a synergic effect between chemotherapy (DOX) and PTT (GO+MnWO_4_) as the components alone were less effective than the combined system: (i) DOX alone was not able to ablate tumor, but the growth rate of tumor volume decreased about 1.5-fold in comparison to the control; (ii) PTT strategy alone (GO+MnWO_4_) had a lower effect than the entire synergic formulation, since it ablated the tumor in 12 days, but it then started to grow until the 16th day. Other GO or NGO based formulations have also used DOX chemotherapy combined with PTT [62,75,76]; however, it was less efficient than GO/MnWO_4_/PEG. A possible explanation for this fact is that in the later formulation, laser treatment was applied twice (6 and 24h post-injection), and the biodistribution assays that quantified Mn indicated that after 24 h post-administration, there was still significant intratumor concentration of the nanocarriers (7% ID/g), thus the second laser irradiation might have potentiated the PTT effect (Table S7 of the supplementary materials)). 

The GO-DOX formulation was the only case of GO-based formulations where DOX was used in a single chemotherapy strategy, and GO was merely a nanocarrier for DOX [71]. GO-DOX formulation was also not able to ablate tumors, but after 14 days of administration, reduced the tumor by approximately 75% [71]. The treatment efficacy also improved 6.5-fold in comparison to free DOX, suggesting a high tumor uptake efficiency of this formulation whose dimensions indicate cell internalization (30 × 6 nm) [71]. Despite not presenting a synergic therapy and not being able to eliminate the tumor, GO-DOX presents the highest loading efficiency of DOX (133.32 wt%) in GBNs, which may account to the good therapeutic outcome reached. Furthermore, authors of this study have evaluated the effect of GO-DOX formulation in angiogenesis, as this is considered a parameter to predict metastasis and to evaluate chemotherapy effect. They concluded that the GO-DOX suppressed 3 times as much tumor angiogenesis when compared with DOX [71]. 

The chemotherapy effect of PTX loaded in NGO based formulation (NGO-PEG-ICG/PTX) was able to promote tumor ablation in 15 days [91]. In this case, PTX was also used as a single chemotherapy strategy, and GO was merely a nanocarrier. Despite not presenting a synergic therapy, this formulation had a small lateral size that assures good tumor cellular uptake, and this was confirmed through biodistribution studies that indicated a significant intratumor concentration of the nanonarriers (29.1% ID/g) 24 h after treatment (Table S7 of the supplementary materials).

Since PTT is not so efficient in GO-based nanosystems as it is in rGO based nanosystems, the success of therapeutic depends largely on combined PTT+PDT or multiple PTT strategies. Except for three studies [47,48,57], all the formulations based in NGO or GO and using a combined PTT+PDT strategy effectively ablated tumors in a short period of time (2 to 14 days) [7,56,67,87,88]. Besides the PTT+PDT synergic combination, the effective cellular uptake of the formulations might be the explanation for their success in tumor ablation. Table S7 of the supplementary materials summarizes the biodistribution information available for some of these formulations than can be correlated to the successful therapeutic outcomes obtained. Two studies that were effective in tumor eradication do not present biodistribution data, so we could not correlate tumor accumulation with the therapeutic outcome [7,88]. However, their efficiency in tumor ablation can be correlated with: (i) the use of synergic PTT+PDT strategy, (ii) their small lateral dimensions that might favor tumor cell uptake, and (iii) the use of two laser irradiation conditions that for sure potentiate the cellular damage via hyperthermia and ROS. Indeed, the formulation GO-PEG-DVDMS possesses a combination of PTT strategy (conferred by NGO) and PDT strategy (conferred by DVDMS) and therapy was achieved upon double irradiation of tumors with a laser of λ = 630 nm (for PDT treatment) and with a laser of λ = 808 nm (for PTT treatment) [88]. Whereas, the formulation NGO-PEG-FA possesses a combination of PTT+PDT strategy (both conferred by NGO) with a laser of λ = 630 nm (for PDT treatment) and with a laser of λ = 808 nm (for PTT treatment) and a laser of λ = 980 nm (for PDT+PTT treatment) [7]. Among the three studies that were not able to ablate tumors, two of them had better performance as the tumors did not grow during the entire time course of the experience (14 days) and at the end of the study the tumor volume in treated animals was 7× [48] or 8× [57] smaller than the tumor volume in the untreated animals (control). Both studies lack biodistribution evaluation and thus it is difficult to analyze the reasons why these formulations were not able to ablate tumors, but we can at least hypothesize that their similar effect of stabilizing tumor growth can be related to the fact of both possessing UCNPs, which, as previously referred, may increase PDT tissue penetration. The other case of a combined PTT and PDT strategy with GO (PheoA+GO:FA-BSA-c-PheoA) was the least successful of all, not being able to avoid tumor growth, although the rate of tumor growth in treated animals was 3× smaller after 14 days than the tumor growth of untreated animals (control) [47]. Authors of this study present biodistribution evaluation and showed that after injection, PheoA+GO:FA-BSA-c-PheoA preferentially accumulated at the tumor (around 2-fold at 3 h), but also accumulated in skin (less than Pheo alone) and the liver [47]. At 24 h post-injection, PheoA+GO:FA-BSA-c-PheoA is only located in the tumor while Pheo is still in the skin. Despite the successful tumor accumulation, there was not a successful tumor ablation as occurred in other studies, and we can hypothesize that this can be related not with the absence of tumor uptake, but with the laser irradiation used. The authors of this study [47] used a single laser for PDT and PTT purposes and this laser is out of the NIR range (λ = 670 nm instead of >800 nm), which is not so adequate to achieve a PTT-effective treatment. It is also worthwhile to mention that another formulation (GO/AuNS-PEG/Ce6) that was able to ablate tumors also used a single irradiation out of the NIR range (λ = 660 nm instead of >800 nm), but in this case, the PTT effect was not only dependent of GO but it was reinforced by the conjugation with AuNS that increases thermal conversion efficiency [87]. Notwithstanding this, the capacity to ablate the tumor using GO/AuNS-PEG/Ce6 took a longer time than the other studies (14 days compared with 2 to 6 days) [56,67]. Moreover, in the treatment with GO/AuNS-PEG/Ce6, the potency of the laser was significantly higher (3 W/cm^2^ for 10 min) and the doses administrated were also significantly higher (10 mg/kg) [87] compared with the treatment with PheoA+GO:FA-BSA-c-PheoA that used a low laser potency of the laser (0.13 W/cm^2^ for 10 min) and a lower dose (2 mg/kg).

Multiple PTT strategies, i.e. PTT effect resultant from the synergy of GO with other components, can be another way of achieving tumor ablation with NGO- or GO-based materials. GO and NGO formulations containing multiple PTT strategies were all able to eradicate tumors in 2 to 6 days [64,73,92]. Besides the PTT synergic combination, the effective cellular uptake of the formulations might be, as stated, the explanation for their success in tumor ablation. Table S7 of supplementary materials summarizes the biodistribution information available for two of these formulations [73,92], and can be correlated to the successful therapeutic outcomes obtained. 

Only one formulation (Au@PLA-(PAH/GO)_n_) containing a multiple PTT strategy (conferred via GO and AuNPs) was not able to ablate tumors, although 9 days after treatment the tumor volume of treated animals was reduced by 80% in comparison with the tumor value of untreated animals (control) [61]. Despite lacking tumor uptake data, this formulation presented very big sizes (1.5 µm), which may explain a small tumor tissue penetration, also explaining the incomplete tumor eradication. It is also worthwhile saying that the efficiency obtained is only possible because: (i) PTT is a type of therapy where the system does not require entering the cell (although being more effective when the cellular uptake occurs); and (ii) the formulation was intratumorally injected instead of intravenously injected, thereby permitting that the big sized micromaterial assessed tumor. Moreover, this study presents a rather short time of evaluation compared with other studies (which always present more than 10 days of evaluation), and an extension of the period of evaluation would be required. Finally, intravenous injection is more applicable to therapy where most tumors are not assessible to intratumoral injection, and in the context of intravenous injection, the bigger sizes of the system administrated suggests some serious toxicity concerns (e.g., by hemolysis) that may shorten animals’ survival. Similarly, another formulation IO/GOCOOH was also intratumorally administrated, and this was for sure a strong reason for the efficient therapeutic outcome obtained (tumor ablation in 2 days) [60]. Thought, in this latter case, the formulation was based in NGO and the lateral dimensions would be adequate for an intravenous administration. 

Regarding GQDs, it seems that there is still room for improving these formulations’ therapeutic efficiency since only two formulations demonstrated capacity to eradicate the tumor. One of these formulations (IR780/GQD-FA) was quite effective (tumor ablation in 6 days) given that it presented a synergic PTT strategy (conferred by GQDs and IR780) that provides the nanosystem with a high photothermal conversion (87.9%), assuring an effective hyperthermia required to kill the cancer cells [65]. The biodistribution studies also corroborate this efficiency by proving IR780/GQD-FA tumor accumulation (Table S7 of the supplementary materials). Another formulation (GQDs) presented a synergic PTT+PDT strategy (PTT conferred by GQDs and PDT conferred by PpIX) and was able to ablate the tumor after 20 days of treatment with two laser irradiations at day 1 and 7 [44]. It is thus assumed that this formulation reached tumor cells, although authors do not present biodistribution data. 

A combined PTT+PDT strategy was also used in cGdots (both strategies conferred by GQDs) and biodistribution studies confirmed that 4 h after injection, GQDs were nonspecifically distributed in the body (liver, lung, and kidneys) and also distributed in the tumor [45]. Despite the observed tumor accumulation, there was no successful tumor ablation and the tumor has grown 1.5× larger in 21 days (though this growth was smaller than that observed in untreated animals). Thus, we can hypothesize that this can be related not with the absence of tumor uptake, but with the laser irradiation used. As stated before, these authors used a single laser for PDT and PTT purposes and this laser is out of the NIR range (λ = 670 nm instead of >800 nm), which is not so adequate to achieve a PTT effective treatment. 

Finally, GQDs were also loaded with DOX in a formulation where DOX was used in a single chemotherapy strategy, and GO was merely a drug nanocarrier [54]. Also, in this case, the tumor is not ablated, and at 23 days after treatment, tumor has grown 6× in size (though this growth was smaller than that observed in untreated animals). No biodistribution studies are presented, thus it is difficult to understand the reason why this formulation is not efficient.

### 4.2. Role of Graphene-Based Nanomaterials in Diagnostic

NGO and GQDs possess appealing optical features that put GBNs in a central role of dye-free labelling diagnostics. Due to a quantum confinement effect that occurs because NGO and GQDs dimensions are smaller than their exciton Bohr radii, the nanosized material possess non-blinking photoluminescence (PL) and photostability [100]. Thus, GQDs fluorescence intensity remains strong under confocal laser illumination and can serve as a reporter guiding the therapeutic nanosystem delivery into cells. Accordingly, the studies reported in this review that were based in GQDs have taken advantage of the PL properties and used it as a diagnostic tool [44,45,49,54,55,63,65,80,82,84,94]. Of additional interest is the fluorescence quenching capability demonstrated by GBNs that result from fluorescence resonance energy transfer (FRET) or non-radiative dipole–dipole interactions between fluorescence species and GBNs. This is being used as an additional diagnostic feature that permits detecting the release of GBNs cargo. Indeed, when GBNs are interacting with the fluorescent cargo (drug or other active) it reduces its fluorescence emission, but the moment the cargo is released, the fluorescence emission is reset. This elegant strategy was used in two studies of this review [54,80].

GBNs photothermal conversion properties can also be applied as a therapy-guiding strategy under a non-labeling IR-TI technique. The application of the NIR laser to induce a PTT effect can be detected using a visible thermal field image (Table S2 of the supplementary materials), which is very interesting due to its non-invasive nature and because it provides real-time imaging. Given that PTT was the therapy modality mostly used by the studies herein revised, IR-TI is, as expected, also widely used in the studies reported [7,8,48,51,56,58,59,60,62,64,65,66,67,69,73,74,77,87,89,92,93] as both strategies are often used together.

The third diagnostic modality most described in the studies revised is PAI, which is also different from GBNs capacity as NIR absorbers or from its easy functionalization with other NIR absorbing materials, such as gold-based nanomaterials (please see Table S3 of the supplementary materials for a more detailed explanation of PAI mechanism). Among the GBNs, rGO has attracted attention as a PAI contrast agent because of its higher NIR absorbance properties. rGO based studies included in this review took advantage of these properties and have used PAI imaging as a diagnostic tool [59,63,74,79]. Notwithstanding the better intrinsic PAI properties of rGO, GO nanomaterials compensate their lower NIR absorption with the higher functionalization capacity. Hence, in some studies herein reviewed, GO nanomaterials were conjugated with other materials as a strategy to enhance NIR absorption and thereby achieved PAI diagnostic modality [56,71,72,73].

In some of the studies reviewed, the unique large surface area and easy functionalization of GBNs has also proved to be very favorable to carry MRI probes [5,48,50,60,80], extending the in vivo half-life of the MRI contrast agent [60]. Moreover, the large molecular weight of GBNs can decrease the rotational motion of the water proton, enhancing T1 and T2 relaxivity and leading to a better image. Similarly, once more due to its large surface area and easy functionalization, GBNs were also explored as a carrier of radionuclides [51].

Finally, GBNs can also take part in Raman imaging or more effectively in diagnostics based in surface enhanced Raman spectroscopy (SERS). Regardless of any treatment that have been used, GBNs normally reveal an appropriate Raman scattering intensity, showing typical bands of D, G, and 2-D characteristics of vibrational modes in the 1000–3000 cm^−1^ range. When functionalized with gold and silver nanoparticles, Raman signals of GBNs are effectively augmented by the surface enhanced Raman scattering. Taking advantage of this strategy, SERS was used to combine microscope cellular imaging with Raman spectroscopy, thereby mapping the presence of GBNs in the cellular tumor tissue [68].

### 4.3. Role of Graphene-Based Nanomaterials in Toxicity

If we can imagine the high surface of GBNs as an advantage for easy interaction with drugs, bioactives, genetic material, and other nanosystems, we can also perceive that the interaction of GBNs with biological structures can be as high, which can be a toxicity concern regarding these highly reactive nanostructures. It has been reported that the GBNs lateral size and thickness, surface charge, colloidal stability, and concentration can strongly impact its biological toxicity [39]. For example, lateral dimensions can dictate the cellular internalization of GBNs (<100 nm) or can promote blood brain barrier permeation (<35 nm), and some reports indicate liver and lung accumulation for larger-sized GBNs [39]. Therefore, the major criticism of the studies included in this study must be the scarce information about the overall nanomaterial size and thickness and surface charge (Table 1). The evaluation of the colloidal stability of the GBNs developed was even more uncommon in the studies reviewed. Additionally, other studies made unsatisfactory evaluations based on the short time stability assessment, such as 3 h, 4 h, 5 h, or 1 day stability [8,50,86]. However, the aggregation nature of less hydrophilic GBNs, such as GO, particularly under physiological conditions (e.g., in serum), can have several consequences and are toxic to different cells [39]. Hence, comprehensive physicochemical characterization is mandatory in future studies of GBNs toxicity.

Colloidal stability of GBNs can be improved by coating their surface with biocompatible materials that enhance aqueous solubility while assuring a better body distribution. PEG coating is one of the most used strategies to reach these aims, and was used as a coating and linker of target ligands in several studies included in this review [5,6,7,46,47,48,49,50,51,53,55,56,58,66,67,71,73,75,76,79,81,85,86,87,88,91] (see Table 1). Other strategies used in the revised studies include coating GBNs with OA [57,60,64], HA [62,69], pDA [59,90], HSA [93], BSA [74], and APGA [77]. OA is a fatty acid that has amphiphilic properties and can be useful to increase the solubility of GO-based nanosystems. HA is biocompatible, increases biodistribution, and can additionally serve to target cancer cells (Table 4). Surface modification with pDA can improve the stability and dispersity of rGOs. An HSA or BSA coating assures increased plasma circulation and wide body distribution. Finally, APGA is a bacteriomimetic coating that assures greater body distribution. 

The in vitro toxicity evaluation made in the studies herein listed also requires some critical observations. Some studies have chosen a cell line representative of healthy tissues to evaluate cytotoxicity while choosing a cancer cell line to evaluate therapeutic outcomes [5,47,48,49,53,57,58,61,62,67,68,70,81,82,85]. Yet, most studies used the same cancer cell line to access toxicity and therapeutic value. However, cancer cell lines can be more resistant or more susceptible according to their genotype. Therefore, primary cell lines would be more advisable to evaluate cytotoxicity [39]. Furthermore, MTT was among the most used methods for cell viability evaluation [5,6,7,44,45,47,48,49,50,53,54,56,57,58,59,60,62,64,66,68,70,73,74,80,82,83,84,85,86,88,90,92,94]. However, MTT has been reported as inadequate for evaluating GBNs cytotoxicity due to a false positive signal. As a replacement to this widely used method, it would be recommended, for instance, to use the trypan blue test, which was only used in three studies [61,82,83]. 

In vivo toxicity evaluation was generally based on the histological observation of healthy tissues or observation of variations in animal weight after treatment has been applied and no toxicity concerns were presented in any of the studies where these evaluations were performed (Table 3). Although many studies have demonstrated bioaccumulation of GBNs in liver and kidneys, few studies have taken toxicity evaluation further by analyzing important liver and kidney function biochemical parameters. Liver function biochemical parameters include the serum quantification of hepatic enzymes such as: alanine aminotransferase (ALT), aspartate transaminase (AST), and alkaline phosphatase (ALP). Kidney function biochemical parameters include the serum quantification of blood urea nitrogen (BUN) and creatinine (CR). Only four studies within the 55 included in this review have made a serum biochemical examination of mice 1 day [77], 7 days [93], 15 days [65], or 50 days [51] after treatment with GBNs. No significant differences were observed in any of these cases in comparison with untreated animals and all the parameters were within the reference values.

Moreover, it is important to carry out the hematological evaluation of: white blood cells, red blood cells, neutrophilic granulocytes, hemoglobin, mean corpuscular hemoglobin concentration, hematocrit, and mean platelet volume and distribution. Once more, only three studies of the 55 included in this review have made this hematological evaluation in mice 15 days [65], 18 days [59], or 50 days [51] after treatment with GBNs. In all these cases, the parameters were within the reference values.

One good indicator of toxicity is the evaluation of inflammatory response after intravenous administration by the quantification of cytokines and tumor necrosis factor α (TNF-α). This evaluation was only made by one study of all the 55 studies included in this review, and the results have demonstrated an inflammatory response as both TNF-α and proinflammatory cytokines were increased after intravenous administrations of GBNs [72].

Finally, all the toxicity assessments performed were based on short-term periods, and a longer period evaluation is necessary.

### 4.4. Pharmaceutical Applications of Graphene-Based Nanomaterials and Clinical Translation Prospects

The potentiality of exploiting GBNs remarkable physical and optical properties for pharmaceutical applications has been pursued over the last few years with interesting scientific endeavors that have proved to be strong contributions in the fields of drug delivery, biosensing, and tissue engineering [101,102,103]. In the field of cancer theranostics, it is particularly important that all the successful research attempts use GBNs to load anticancer drugs, bioactives, or nucleic acids in synergic applications of chemotherapy, gene therapy, and photothermal/photodynamic therapy [101,102,103]. Moreover, in comparison with highly toxic inorganic quantum dots used for theranostic purposes, the ongoing studies indicate a general satisfactory biocompatibility of GBNs and even when harmful effects were detected there is a sturdy proof that GBNs toxicity can be modulated and is reliant on specific applications of this nanomaterial [104]. Notwithstanding these satisfactory signs that GBNs can be improved to safer forms, there is still a lack of standardization to assure this control [103,104]. Consequently, the European Scientific Committee on Emerging and Newly Identified Health Risks has included graphene in the classification of dangerous substances, stating that there are still many risk related knowledge gaps to be filled before considering this nanomaterial safe [105]. This is sadly what happens with most nanomaterials. Indeed, the toxicity grade for a material can be roughly controlled by three factors (composition, concentration, and exposure route), whereas for a nanomaterial the toxicity grade relies on over 10 factors, and it is much more difficult to control these factors or to report them in a standardized way, such that they can be compared and improved [106]. 

Therefore, despite the attention-grabbing results obtained to this point and the evident success, there are still several issues to unravel before a clinical translation is possible. The main issues include: (i) the dearth of standardization within the production of the different GBNs types; (ii) the lack of thorough characterization to control lateral size, aggregation state (single vs. few layers of graphene), colloidal stability, and oxidation grade; (iii) the presence of remnant contaminants resultant from production processes that may add toxicity to GBNs; (iv) the need to understand how dimensions and surface chemistry affect cellular uptake or physical adsorption to biomolecules like membranes and proteins; (v) the necessity to evaluate the generation of ROS and its consequences in healthy tissues; (vi) the scarce number of biodistribution studies; and (vii) the need to evaluate toxicity in a wider context that involves not only cytotoxicity studies but also genotoxicity, biodegradation, distribution, and accumulation into organs, metabolism, and evaluation upon long term exposure. If these main issues are not addressed, the GBNs developed will have trouble fulfilling the rigid criteria for translation of novel nanomaterials into significant clinical uses. 

Efforts are being made by different scientists to establish important guidelines, tutorials, or to define challenges that must be pursued to develop low-toxicity and safer nanomaterials that can be accepted by regulatory authorities. Although being out of the scope of this review, these attempts of standardization deserve special attention by those that work with GBNs for pharmaceutical applications and wish to translate their studies into a clinical use as theranostics (for a more extensive discussion, please refer to good reviews on the subject [103,104,106]).

Although the translation of GBNs to a clinical context of pharmaceutical applications is still very far from being possible, it is worth mentioning that the enthusiasm of the scientific community with the GBNs breakthroughs is shared by the National Institutes of Health (NIH) and its National Institute of Biomedical Imaging and Bioengineering (NIBIB) program. Indeed, this program is focused on coordinating engineering and physical sciences with life sciences to propel fundamental research and medical care and has supported studies included in this review [66,71,88] through the Intramural Research Program, which indicates an interest in the innovative diagnosis and therapy strategies provided by GBNs.

## Figures and Tables

**Table 1:** Summary of characterization features of GBNs, type of graphene nanomaterial, and inclusion criteria based on reported therapeutic and/or diagnostic role of graphene in the overall system developed. The figures mentioned in this table can be found in each referenced study and represent examples from the revised studies where therapeutic and/or diagnostic relevance of graphene is clearly illustrated.

Graphene-Based Nanomaterial	Graphene Type	Coating	Theranostic System Size (D or D × T)	Zeta-Potential/ Colloidal Stability in Serum	Proven Graphene Therapeutic Relevance	Proven Graphene Diagnostic Relevance	Ref.
Au@PLA-(PAH/GO)n	GO		1.5 µm	n.r.		USI (Figure 2) s.e. Au@PLA	[61]
UCNPs-NGO/ZnPc	GO	PEG	≈ 300 × 1.5 nm (GO) + 40 nm (UCNPs)	n.r.	PTT (Figure 3 and Figure S4)		[85]
GO-HA-Ce6	GO		≈ 440 nm	n.r. 1 d stability	PTT (Figure 3) s.e. Ce6		[52]
ICG-GO-FA	GO	PEG-FA	≈ 200 nm	n.r. 3 h stability	PTT (Figure S3, Figure 2 and Figure 3) s.e. ICG		[86]
GO-AuNS	GO		≈ 0.8 μm	−19 mV 10 days	PTT (Figure 6) s.e. AuNS		[70]
GQD-Cur	GO		≈ 150 nm (GQD-Cur) 3–6 nm (GQD)	n.r.		PL (Figure 6)	[78]
GO-Abs/Cy7	GO		100–600 × 1.2 nm	n.r.	PAT (Figure 3 and Figure 9) s.e. Cy7	PAI (Figure 3 and Figure 6) s.e. Cy7	[72]
GDH	GO	HA	≈ 120 nm (GDH) ≈ 30 nm (GO)	n.r. 7 days	PTT (Figure 5a)		[62]
PheoA+GO:FA-BSA-c-PheoA	GO	PEG-FA	≈ 180 nm	≈ −30 mV n.r.	PTT + PDT (Figure 7 and Figure 9) s.e. Pheo		[47]
GO-PEG-ZnS:Mn-DOX	GO	PEG-ZnS:Mn	n.r.	n.r.		PL (Figure 4) s.e. ZnS:Mn	[53]
CPGA	GO	PEG	≈ 230 × 15 nm	≈ −25 mV 7 d stability	PTT (Figure 3) s.e. Au and Cy5.5	PAI (Figure 2) FI (Figure 2) s.e. Au	[56]
GO@Gd-PEG-FA/DOX	GO	PEG-FA	n.r	≈ −6 mV	PTT (Figure 6)		[75]
GO/AuNS-PEG/Ce6	GO	PEG	≈ 400 × 18 nm	≈ −38 mV 1 d stability	PTT (Figure 2b) s.e. AuNS		[87]
GO/Bi_2_Se_3_/PVP	GO		≈ 150 nm	≈ −18 mV (GO) n.r.	PTT (Figure 5d) s.e. Bi_2_Se_3_		[92]
GO/UCNPs ZnFe_2_O_4_	GO	PEG	≈ 400 nm	≈ −17 mV n.r.	PTT (Figure 2d) PDT+PTT (Figure 3) s.e. UCNPs		[48]
GO/MnWO_4_/PEG	GO	PEG	≈ 130 nm	≈ −26 mV n.r.	PTT (Figure 3)		[5]
LOGr-Pc-LHRH	LOGr	PEG	≈ 80 nm	n.r. n.r.	PTT (Figure 3a and Figure 6b) PDT (Figure 6c) s.e. Pc		[81]
GO-DOX	NGO	PEG	≈ 30 × 6 nm	n.r.		PAI (Figure 5) s.e. Cy 5.5	[71]
ICG-FeCl_3_@GO	NGO	_	≈ 40 nm	n.r.	PTT (Figure 3d) s.e. ICG		[83]
GO@Ag-DOX-NGR	NGO	DSPE-PEG-NGR	≈ 40 nm	−29 mV	PTT (Figure 6) s.e. Ag		[76]
GO-PEG-DVDMS	NGO	PEG	≈ 20.5 × 1.5 nm	n.r.	PTT (Figure 2a, Figure 3a and Figure 3d) s.e. DVDMS		[88]
IO/GO-COOH	NGO	OA	≈ 50 × 20 nm	n.r.	PTT (Figure 5) s.e. IO	MRI (Figure 3) s.e. IO	[60]
GO-PEG-CysCOOH	NGO	PEG-CysCOOH	< 50 × 2 nm	n.r. 1 d stability	PTT (Figure 2 and Figure 4) s.e. CysCOOH	PAI (Figure 3) s.e. CysCOOH	[73]
Au@NGO	NGO		≈ 98 nm	≈ −28 mV n.r.		SERS (Figure 3) s.e. Au	[68]
NGO-PEG-FA	NGO	PEG-FA	≈ 100 nm	n.r	PTT (Figure 4, Figure 5, Figure 6 and Figure 7	FI (Figure 3)	[7]
NGO-IR-808	NGO	PEG	≈ 20–40 × 3 nm	n.r.	PTT (Figure 2, Figure 3 and Figure 7) s.e. IR-808	FI (Figure 4 and Figure 6) IR-TI (Figure 7) s.e. IR-808	[67]
NGO-PEG-ICG/PTX	NGO	PEG-ICG	< 100 × 1 nm	≈ −30 mV 14 d stability	PTT (Figure 4, Figure S5 and Figure 6) s.e. ICG	FI (Figure 5) s.e. ICG	[91]
NGO-UCNPs-Ce6	NGO	OA	≈ 100 nm (GO)+ 48 nm (UCNPs)	n.r.	PTT (Figure S4, Figure 7 and Figure 8) PDT+PTT (Figure 3, Figure 7 and Figure 8) s.e. UCNPs		[57]
UCNP@NGO	NGO	OA	≈ 100 nm (GO)+ 55 nm (UCNPs)	n.r.	PTT (Figure 2, Figure 4 and Figure 5) s.e. UCNPs		[64]
BSA/nano-rGO	rGO	BSA	≈ 70 nm	≈ −30 mV 30 d stability	PTT (Figure 3)	PAI (Figure 4 and Figure 5)	[74]
rGONM-PEG-Cy7-RGD	rGO	PEG	≈ 61 nm	n.r.	PTT (Figure 2)		[46]
rGO-Fe_2_O_3_@AuNPs	rGO	NH_2_-PEG	≈ 610 nm	−21.1 mV > 5 h	PTT (Figure 3a) s.e. Fe_2_O_3_@Au NPs		[50]
rGO nanosheets	rGO	HA	≈ 115 nm	≈ −60 mV n.r.	PTT (Figure 4a and Figure 5a) s.e. ICG		[69]
^131^I-RGO-PEG	rGO	PEG	≈ 50 × 3.5 nm	n.r. 7 days	PTT (Figure 2) s.e. ^131^I	γ-image (Figure 3) s.e. ^131^I	[51]
rGO-AuNRVe	rGO	PEG	≈ 74 nm	n.r.	PTT (Figure 2) s.e. AuNR	PAI (Figure 5) s.e. AuNR	[79]
anti-EGFR-PEG-rGO@ CPSS-Au-R6G	rGO	PEG-Anti-EGFR	< 200 nm	n.r.	PTT (Figure S5 and Figure 7b) s.e. AuNPs		[6]
ICG-PDA-rGO	rGO	PDA	1–5 μm × 1 nm	n.r.	PTT (Figure 3 and Figure 8) s.e. ICG	PAI (Figure 4 and Figure 6) IR-TI (Figure 7) s.e. ICG	[59]
rGO-GSPs	rGO	PEG	≈ 100 nm	n.r. 3 d stability	PTT (Figure 2a and Figure 2b) s.e. AuNPs	PAI (Figure 2d and Figure 2e) s.e. AuNPs	[66]
rGO-mfHSA	rGO	mfHSA	≈ 200 nm	−25 mV 2 d stability	PTT (Figure 3c)		[93]
FA-PEG-Lip@rGO/Res	rGO	PEG-FA	≈ 220 nm	≈ −24 mV 7 d stability	PTT (Figure 7b)	IR-TI (Figure 7a)	[58]
ArGO	rGO	APGA	≈ 115 nm	≈ −38 mV 28 d stability	PTT (Figure 5b and Figure 6a)	IR-TI (Figure 5a)	[77]
AAP10-pDA/rGO	rGO	pDA	300 nm × 3.5 nm	n.r.	PTT (Figure 2) s.e. pDA		[90]
cGdots	GQDs		≈ 5 nm	n.r. n.r.	All therapeutic outcomes came from GQDs	All diagnostic outcomes came from GQDs	[45]
GQDs	GQDs		2–6 nm	n.r.	All therapeutic outcomes came from GQDs	All diagnostic outcomes came from GQDs	[44]
PLA-PEG-grafted GQDs (f-GQDs)	GQDs	PLA-PEG	≈ 22 × 1.7 nm	≈ −4 mV n.r.		PL (Figure 4)	[55]
AS1411@GQD	GQDs			≈ −13 mV n.r.	PTT (Figure 6a and Figure 6b) s.e. AS1411		[84]
HA-GQD -SiO_2_ NPs	GQDs		≈ 40 nm	n.r.	PDT (Figure 6) s.e. HA and SiO_2_		[94]
GQDs@Cys-BHC	GQDs		≈ 5 nm	n.r.		FI (Figure 8)	[82]
Fe_3_O_4_@SiO_2_@GQDs-FA/DOX	GQDs		≈ 25 nm	≈ −18 mV n.r.		PL (Figure 3) FI (Figure 5)	[80]
GQD-MSN--DOX	GQDs		50-80 nm	n.r.	PTT (Figure 6 and Figure 9)		[89]
GQD-PEG-P	GQDs	PEG	8 × 1 nm	≈ + 14 mV n.r.	PTT (Figure 3)	FI (Figure 5)	[49]
DOX@GQD-P-Cy	GQDs		≈ 15 nm	≈ −4 mV (w/o) DOX n.r.		FI (Figure 4d–e) s.e. DOX by FRET	[54]
DL-GQD-comp	GQDs		≈ 220 nm	n.r.	―	PL (Figure 5)	[63]
IR780/GQDs-FA	GQDs		8.5 × 1.5 nm	n.r.	PTT (Figure 5a and Figure 5b) s.e. IR780	FI (Figure 6) IR-TI (Figure 7a) s.e. IR780	[65]
SCNA (DOX/GQD)	GQDs	HTPGS	< 5 nm	n.r. 4 h stability	PTT (Figure 5e)	CLSMI (Figure 4)	[8]

Table 1 abbreviations: GO—Graphene oxide; NGO—Nanographene oxide; rGO—Reduced Graphene oxide; GQDs—Graphene Quantum Dots; D—Diameter; D × T—Diameter × Thickness; d–day(s); n.r.—not reported; s.e.—synergic effect with; CLSMI—confocal laser scanning microscopy imaging; FI—fluorescence imaging; FRET—fluorescence resonance energy transfer; IR-TI—infrared thermal imaging; MRI—magnetic resonance imaging; PAI—photoacoustic imaging; PAT—photoacoustic therapy; PDT—photodynamic therapy; PL—Photoluminescence; PTT—photothermal therapy; SERS—Super Enhanced Raman Spectroscopy; USI—ultra sound imaging. AAP10—Antiarrhythmic peptide 10 (promotes bystander effect); Abs—integrin α_v_β_3_ mAb (targeting ligand); Ag—silver; anti-EGFR—anti-epidermal growth factor receptor (targeting ligand); APGA—amphiphilic poly-γ-glutamic acid; ArGO—rGO coated with amphiphilic poly-γ-glutamic acid; AS1411—aptamer of 26-base guanine-rich short oligonucleotide (targeting ligand); Au—gold; AuNPs—gold nanoparticles; AuNRVe—gold nanorod vesicles; AuNR—Gold nanorods; AuNS—Gold nanostars; Bi_2_Se_3_—Bismuth Selenide; BHC—Berberine hydrochloride; BSA—bovine serum albumin; Ce6—Chlorin e6 (photosensitizer); cGdots—carboxylated graphene dots; Cy5.5—Cyanine 5.5 (NIR dye and photosensitizer); Cy7—Cyanine 7 (NIR dye and photosensitizer); Cys—Cysteamine hydrochloride (NIR dye); Cys-COOH—Cysteine- rich Carboxy-terminal domain CPGA—theranostic probe formed by Cy5.5 (NIR dye) labelled-matrix metalloproteinase-14 (MMP-14) substrate (CP) conjugated onto the GO/Au complex (GA); CPSS—carbon porous silica nanosheets; Cur—curcumin; DL-GQD-comp—doxorubicin hydrochloride loaded GQD complex; DOX—doxorubicin hydrochloride; DSPE—1,2-distearoyl-*sn*-glycero-3-phosphoethanolamine; DVDMS—bis[1-[6,7-bis[2-(sodium carbonate ethyl]-1,3,5,8,-tetramethyl-2-vinyl-porphin-4-yl]ethyl]ether (photosensitizer); FA—Folic acid (target ligand); FeCl_3_—Iron chloride; Fe_2_O_3_ and Fe_3_O_4_—Iron oxide nanoparticles; Gd—Gadolinium; GDH—Graphene–DOX conjugate in HA nanogel; GSPs—gold superparticles; HA—hyaluronic acid (target ligand); HA-GQD—complex of Hypocrellin A (photosensitizer), HA and GQD; HTPGS—N-acetyl histidine-functionalized D-α-tocopherol polyethylene glycol 1000 succinate; ^131^I—Iodine-131 (radioisotope); ICG—NIR fluorescence dye; IO—iron oxide; IR780—IR780 iodide (NIR dye); IR-808—Heptamethine indocyanine dye (photosensitizer); LHRH—luteinizing hormone-releasing hormone peptide; Lip—Phospholipids; LOGr—low-oxygen graphene; mfHSA—multifunctional human serum albumin—HSA functionalized with indocyanine green (ICG) and lactobionic acid (LA); MnWO_4_—manganese tungstate; MSN—mesoporous silica nanoparticles; NGR—Asn-Gly-Arg peptide (target ligand); OA—Oleic acid; P—porphyrin; PAH—poly (allylamine hydrochloride); Pc—phthalocyanine; P-Cy—Cyanine 5.5 dye conjugated to GQD though a cathepsin D-responsive peptide (P); PDA or pDA—Polydopamine (reduces GO improves water solubility and biocompatibility and increases NIR absorption); PEG—Polyethylene glycol; PheoA—Pheophorbide A (photosensitizer); PLA—polylactic acid; PTX—Paclitaxel; PVP—polyvinylpyrrolidone; R6G—Rhodamine 6G; Res—Resveratrol; rGONM—reduced graphene oxide nanomesh; RGD—arginine–glycine–aspartic acid-based peptide (target ligand); SCNA—size-changeable graphene quantum dot nanoaircraft; SiO_2_ NPs—silicon dioxide nanoparticles; UCNPs—upconversion luminescence nanoparticles; ZnFe_2_O_4_—Zinc ferrite nanoparticles; ZnPc—Zinc phthalocyanine (photosensitizer); ZnS:Mn—manganese-doped zinc sulfide nanoparticles.

**Table 2:** Theranostic strategies of graphene-based nanomaterials containing GO, NGO, rGO and GQDs.

Graphene-Based Nanomaterial	THERAPY					DIAGNOSTIC										Ref.
	Drug, Bioactive, PS, or Gene	Trigger	MHT	PTT PAT	PDT	Sensing/ Targeting	Therapy Guiding									
							Optical					Non-Optical				
							FI, PL	2PFI or UCLI	IR-TI	Raman, SERS	X-ray	MRI	PET, CT SPECT	PAI	USI	
**Theranostic strategies of graphene-based nanomaterials containing GO**																
Au@PLA-(PAH/GO)_n_				+									+		+	[61]
UCNPs-NGO/ZnPc	ZnPc (PS)			+	+			+								[85]
GO-HA-Ce6	Ce6 (PS)	HA		+	+		+									[52]
ICG-GO-FA				+		FA	+							+		[86]
GO-AuNS				+						+						[70]
GQD-Cur	Cur (Bioactive)						+									[78]
GO-Abs/Cy7				+PAT		Abs								+		[72]
GDH	DOX (Drug)	NIR, pH GDH		+		HA	+		+							[62]
PheoA+GO:FA-BSA-c-PheoA	PheoA (PS)	pH		+	+	FA	+									[47]
GO-PEG-ZnS:Mn DOX	DOX (Drug)	pH					+									[53]
CPGA	Cy5.5 (PS)			+	+	MMP-14(P)	+		+					+		[56]
GO@Gd-PEG-FA/DOX	DOX (Drug)			+		FA						+				[75]
GO/AuNS-PEG/Ce6	Ce6 (PS)			+	+				+							[87]
GO/Bi_2_Se_3_/PVP				+					+				+	+		[92]
GO/UCNPs ZnFe_2_O_4_	ZnFe_2_O_4_ (PS)			+	+			+	+			+	+	+		[48]
GO/MnWO_4_/PEG	DOX (Drug)	NIR, pH		+								+		+		[5]
LOGr-Pc-LHRH	Pc (PS)	NIR		+	+		+									[81]
**Theranostic strategies of graphene-based nanomaterials containing NGO**																
GO-DOX	DOX (Drug)	pH												+		[71]
ICG-FeCl_3_@GO	ICG (PS)			+	+	AGE-Aptamer	+									[83]
GO@Ag-DOX-NGR	DOX (Drug)	NIR		+		NGR					+					[76]
GO-PEG-DVDMS	DVDMS (PS)	NIR		+	+									+		[88]
IO/GO-COOH				+					+			+				[60]
GO-PEG-CysCOOH				+					+					+		[73]
Au@NGO	DOX (Drug)										+					[68]
NGO-PEG-FA				+	+	FA	+		+							[7]
NGO-IR-808	IR-808 (PS)	NIR		+	+	BPEI	+		+							[67]
NGO-PEG-ICG/PTX	PTX (Drug)	pH					+									[91]
NGO-UCNPs-Ce6	Ce6 (PS)			+	+			+								[57]
UCNP@NGO				+				+	+							[64]
**Theranostic strategies of graphene-based nanomaterials containing rGO**																
BSA/nano-rGO				+					+					+	+	[74]
rGONM-PEG-Cy7 RGD				+		RGD	+									[46]
rGO-Fe_2_O_3_ @AuNPs	DOX (drug)	NIR Magnetic		+			+					+				[50]
rGO nanosheets				+					+							[69]
^131^I-rGO-PEG	^131^I (Radio- therapy)	NIR		+					+				+			[51]
rGO-AuNRVe	DOX (drug)	NIR pH		+									+	+		[79]
anti-EGFR-PEG-rGO@CPSS-Au-R6G				+		Anti-EGFR	+			+						[6]
ICG-PDA-rGO				+					+					+		[59]
rGO-GSPs				+					+					+		[66]
rGO-mfHSA				+					+							[93]
FA-PEG-Lip@rGO/Res	Res (Bioactive)			+		FA			+							[58]
ArGO				+					+							[77]
AAP10-pDA/rGO	AAP10 (Peptide)			+					+							[90]
**Theranostic strategies of graphene-based nanomaterials containing GQDs**																
cGdots				+	+		+									[45]
GQDs	PpIX (PS)				+		+									[44]
PLA-PEG-grafted GQDs (f-GQDs)	IP ASODN (Gene)						+									[55]
AS1411@GQD	AS1411 (Gene)	NIR		+		AS1411	+									[84]
HA-GQD-SiO_2_ NPs	Hypocrellin (PS)				+		+									[94]
GQDs@Cys-BHC	BHC (Bioactive)	pH					+									[82]
Fe_3_O_4_@SiO_2_ @GQDs-FA/DOX	DOX (Drug)	pH				FA FRET	+					+				[80]
GQD-MSN-DOX	DOX (Drug)			+					+							[89]
GQD-PEG-P	P (PS)			+	+		+									[49]
DOX@GQD--P-Cy	DOX (Drug)	Peptide P				FRET	+									[54]
DL-GQD-comp	DOX (Drug)	pH				HER	+									[63]
R780/GQDs-FA				+		FA	+		+							[65]
SCNA (DOX/GQD)	DOX (Drug)	NIR + HTPGS		+			+		+							[8]

Table 2 abbreviations: GO—Graphene oxide; NGO—Nano Graphene oxide; rGO—Reduced Graphene oxide; GQDs—Graphene Quantum Dots; PS—photosensitizer; CT—computed tomography; SPECT—Single Photon Emission Computed Tomography; FI—fluorescence imaging; 2PFI—two photon fluorescence microscopy imaging; FRET—fluorescence resonance energy transfer; IR-TI—infrared thermal imaging; MHT—magnetic hyperthermia therapy; MRI—magnetic resonance imaging; PAI—photoacoustic imaging; PAT—photoacoustic therapy; PDT—photodynamic therapy; PET—positron emission tomography; PL—Photoluminescence; PTT—photothermal therapy; SERS—Super Enhanced Raman Spectroscopy; UCLI—upconversion luminescence imaging; USI—ultrasound image; AAP10—Antiarrhythmic peptide 10 (promotes bystander effect); Abs—integrin α_v_β_3_ mAb (targeting ligand); Ag—silver; AGE-aptamer—targets melanoma inhibitor of apoptosis protein (ML-IAP) overexpressed in melanoma cells; anti-EGFR—anti-epidermal growth factor receptor (targeting ligand); APGA—amphiphilic poly-γ-glutamic acid; ArGO—rGO coated with amphiphilic poly-γ-glutamic acid; AS1411—aptamer of 26-base guanine-rich short oligonucleotide (targeting ligand); ASODN—survivin antisense oligodeoxynucleotide; Au—gold; AuNPs—gold nanoparticles; AuNRVe—gold nanorod vesicles; AuNR—Gold nanorods; AuNS—Gold nanostars; Bi_2_Se_3_—Bismuth Selenide; BHC—Berberine hydrochloride; BPEI—Branched polyethylenimine; BSA—bovine serum albumin; Ce6—Chlorin e6 (photosensitizer); cGdots—carboxylated graphene dots; Cy5.5—Cyanine 5.5 (NIR dye and photosensitizer); Cy7—Cyanine 7 (NIR dye and photosensitizer); Cys—Cysteamine hydrochloride (NIR dye); Cys-COOH—Cysteine- rich Carboxy-terminal domain CPGA—theranostic probe formed by Cy5.5 (NIR dye) labelled-matrix metalloproteinase-14 (MMP-14) substrate (CP) conjugated onto the GO/Au complex (GA); CPSS—carbon porous silica nanosheets; Cur—curcumin; DL-GQD-comp—doxorubicin hydrochloride loaded GQD complex; DOX—doxorubicin hydrochloride; DSPE—1,2-distearoyl-*sn*-glycero-3-phosphoethanolamine; DVDMS—bis[1-[6,7-bis[2-(sodium carbonate ethyl]-1,3,5,8,-tetramethyl-2-vinyl-porphin-4-yl]ethyl]ether (photosensitizer); FA—Folic acid (target ligand); FeCl_3_—Iron chloride; Fe_2_O_3_ and Fe_3_O_4_—Iron oxide nanoparticles; Gd—Gadolinium; GDH—Graphene–DOX conjugate in HA nanogel; GSPs—gold superparticles; HA—hyaluronic acid (target ligand); HA-GQD—complex of Hypocrellin A (photosensitizer), HA and GQD; HER—Herceptin, monoclonal antibody that targets HER2 Positive Metastatic Breast Cancer (target ligand); HTPGS—N-acetyl histidine-functionalized D-α-tocopherol polyethylene glycol 1000 succinate; ^131^I—Iodine-131 (radioisotope); ICG—NIR fluorescence dye; IO—iron oxide; IR780—IR780 iodide (NIR dye); IR-808—Heptamethine indocyanine dye (photosensitizer); LHRH—luteinizing hormone-releasing hormone peptide; Lip—Phospholipids; LOGr—low-oxygen graphene; mfHSA—multifunctional human serum albumin—HSA functionalized with indocyanine green (ICG) and lactobionic acid (LA); MMP-14(P)—Peptide substrate of MMP-14, a key endopeptidase that is overexpressed on tumor cell surface; MnWO_4_—manganese tungstate; MSN—mesoporous silica nanoparticles; NGR—Asn-Gly-Arg peptide that can selectively recognize CD13 isoform selectively overexpressed in tumor vasculature and certain tumor cells (target ligand); OA—Oleic acid; P—porphyrin; PAH—poly (allylamine hydrochloride); Pc—phthalocyanine; P-Cy—Cyanine 5.5 dye conjugated to GQD though a cathepsin D-responsive peptide (P); PDA or pDA—Polydopamine (reduces GO improves water solubility and biocompatibility and increases NIR absorption); PEG—Polyethylene glycol; PheoA—Pheophorbide A (photosensitizer); PLA—polylactic acid; PpIX—protoporphyrin IX (photosensitizer); PTX—Paclitaxel; PVP—polyvinylpyrrolidone; R6G—Rhodamine 6G; Res—Resveratrol; rGONM—reduced graphene oxide nanomesh; RGD—arginine–glycine–aspartic acid-based peptide (target ligand); SCNA—size-changeable graphene quantum dot nanoaircraft; SiO_2_ NPs—silicon dioxide nanoparticles; UCNPs—upconversion luminescence nanoparticles; ZnFe_2_O_4_—Zinc ferrite nanoparticles; ZnPc—Zinc phthalocyanine (photosensitizer); ZnS:Mn—manganese-doped zinc sulfide nanoparticles.

**Table 3:** Therapeutic outcomes and toxicity evaluation of graphene-based nanomaterials containing GO, NGO, rGO and GQDs.

Graphene-Based Nanomaterial (GBNs)	In Vitro					In Vivo					Ref
	Cell Model	Dark Cell Viability [GBNs] (Method)	NIR Laser	Drug or Active, Dose, AL(wt%)	Therapeutic Outcomes [GBNs] (Method)	Animal Model and Dose	Toxicity (Method)	NIR Laser	Drug or Active, Dose, AL(wt%)	Therapeutic Outcomes (Method)	
**Therapeutic outcomes and toxicity evaluation of graphene-based nanomaterials containing GO**											
Au@PLA-(PAH/GO)n	HeLa HUVECs	90% HUVECs viability with 1000 µg/mL (Trypan blue)	λ = 808 nm P = 6.67 W/cm^2^ t = 6 min	―	ΔT = 21 °C 0.1 < IC_50_ < 0.25 µg/mL >80% HeLa cell death with 0.5 µg/mL (Trypan blue)	Xenograft mice (HT1080 cells) 20 mg/Kg	Minimal effects (body weight)	λ = 808 nm P = 2.23 W/cm^2^ t = 10 min	―	ΔT = 18.6 °C Tumor reduced ≈80% in 5d (volume)	[61]
UCNPs-NGO/ZnPc	KB HeLa	>90% with 320 µg/mL (MTT)	- PDT: λ = 630 nm P = 50 mW/cm^2^ t = n.r. - PTT: λ = 808 nm P = 2 W/cm^2^ t = 10 min	ZnPc n.r.	ΔT = 23 °C 40 < IC_50_ < 80 µg/mL 90% cell death with 320 µg/mL (MTT)	―	―	―	―	―	[85]
GO-HA-Ce6	A549	≈84% with 1.8 µM Ce6 (CCK-8)	λ = 810 nm P = 4 W/cm^2^ t = 8 min	―	ΔT = 27 °C IC_50_ n.r. 90% cell death with 1.8 µM Ce6 (CCK-8)	―	―	―	―	―	[52]
ICG-GO-FA	HeLa	≈100% with 20 µg/mL (MTT)	λ = 808 nm P = 2 W/cm^2^ t = 10 min	―	ΔT ≈ 40 °C IC_50_ n.r. >90% cell death with 20 µg/mL (MTT)	―	―	―	―	―	[86]
GO-AuNS	SKBR-3 MCF-10a	≈95% in SKBR-3 and MCF-10a with 40 µg/mL (MTT)	λ = 808 P = 0.75 W/cm^2^ t = 2	―	ΔT = 60 °C IC_50_ n.r. 81% cell death with 10 µg/mL (MTT)	―	―	―	―	―	[70]
GQD-Cur	HCT166	>90% with 100 µg/mL (SRB)	―	Cur AL ≈ 41%	ΔT = 18 °C 6 < IC_50_ < 12.5 µg/mL >90% cell death with 100 µg/mL (SRB)	Xenograft mice (HCT166) 10 mg/Kg	Minimal effects (body weight) Undetectable tissue damage (histological)	―	Cur AL ≈ 41%	Tumor growth reduced 4× in 14 d (volume)	[78]
GO-Abs/Cy7	U87-MG	≈95% with 42.8 µg/mL (CCK-8)	λ = n.r. P = 0.016W/cm^2^ t = n.r.	―	5.3 < IC_50_ < 10.7 µg/mL >90% cell death with 16.05 µg/mL (CCK-8)	Xenograft mice (U87-MG cells) 43.4 µg/mouse	Minimal effects (body weight) Minimal tissue damage (histological)	λ = 753 nm P = 0.02 W/cm^2^ t = 1 min	―	Tumor did not grow in 20 d and was 7× smaller than control Survival ≥ 20 d Noticeable tumor tissue damage (histological)	[72]
GDH	MDCK A549	≈80–85% MDCK cell viability with 100 µg/mL (MTT)	λ = 670 nm P = 1 W/cm^2^ t = 5 min	DOX 100 μg/mL AL n.r.	ΔT ≈ 30 °C IC_50_ = 50 µg/mL 70–75% A549 cell death with 100 µg/mL (MTT)	Xenograft mice (A549 cells) 2.5 mg/Kg	Minimal effects (body weight) Minimal tissue damage (histological)	λ = 670 nm P = 1 W/cm^2^ t = 30 min	DOX n.r.	ΔT ≈ 17 °C Tumor reduced ≈40% in 18 d (volume) Noticeable tumor tissue damage (histological)	[62]
PheoA+GO:FA-BSA-c-PheoA	B16F10 MCF-7	100% with 0.375 µg/mL (MTT)	λ = 670 nm P = 0.13 W/cm^2^ t = 10 min	―	ΔT = 6 °C IC_50_ < 0.375 µg/mL 95% cell death with 1.5 µg/mL (MTT)	Xenograft mice (B16F10 cells) 2 mg/Kg	Minimal effects (body weight)	λ = 670 nm P = 0.13 W/cm^2^ t = 10 min	―	Tumor growth reduced 3× in 14 d (volume)	[47]
GO-PEG-ZnS:Mn-DOX	HeLa; CHO-K1	100% with 1000 µg/mL (w/o DOX) (MTT)	―	DOX 300 μg/mL AL ≈ 10%	IC_50_ < 2.5 µg/mL 85% cell death with 10 µg/mL (MTT)	―	―	―	―	―	[53]
CPGA	SCC7	≈70% with 200 µg/mL (MTT)	λ = 808 nm P = 0.3 W/cm^2^ t = 10 min	―	ΔT = 23 °C 50 < IC_50_ <100 µg/mL 80% cell death with 200 µg/mL (MTT)	Xenograft mice (SCC7 cells) 10 mg/Kg	Minimal effects (body weight)	λ = 808 nm P = 0.75 W/cm^2^ t = 10 min	―	ΔT = 15.9 °C Tumor abl. in 6 d (volume)	[56]
GO@Gd-PEG-FA/DOX	MCF-7	≈85% with 10 µg/mL (w/o DOX) (SRB)	λ = 808 nm P = 2.5 W/cm^2^ t = 5 min	DOX 10 µg/mL AL≈94%	ΔT = 22.1 °C IC_50_ n.r. 80% cell death with 10 µg/mL (SRB)	Xenograft mice (S180 cells) 10 mg/Kg	Minimal effects (body weight)	λ = 808 nm P = 2 W/cm^2^ t = 5 min	DOX 5 mg/kg AL ≈ 94%	ΔT = 9.6 °C Tumor growth reduced 7× in 12 d (volume) Noticeable tumor tissue damage (histological)	[75]
GO/AuNS- -PEG/Ce6	EMT6	>95% with 100 µg/mL (CCK-8)	λ = 660 nm P = 2 W/cm^2^ t = 8 min	―	ΔT = 50 °C IC_50_ < 0.375 µg/mL ≈80% cell death with 150 µg/mL (CCK-8)	Xenograft mice (EMT6 cells) 10 mg/kg	Minimal effects (body weight) Minimal tissue damage (histological)	λ = 660 nm P = 3 W/cm^2^ t = 10 min	―	ΔT = 20 °C Tumor abl. in 14 d (volume) Noticeable tumor tissue damage (histological)	[87]
GO/Bi_2_Se_3_/ PVP	HeLa	>90% with 75 µg/mL (MTT)	λ = 808 nm P = 0.3 W/cm^2^ t = 10 min	―	ΔT = 33 °C 25 < IC_50_ < 50 µg/mL ≈90% cell death with 100 µg/mL (MTT)	Xenograft mice (HeLa) 0.2 mg/mouse	Minimal effects (body weight) Minimal tissue damage with 0.4 mg/mouse (histological)	λ = 808 nm P = 0.4 W/cm^2^ t = 5 min	―	ΔT = 23 °C Tumor abl. in 2 d (volume) Noticeable tumor tissue damage (histological)	[92]
GO/UCNPs ZnFe_2_O_4_	HeLa; L929	80% with 500 µg/mL (MTT)	λ = 980 nm P = 0.8 W/cm^2^ t = 15 min	―	ΔT ≈ 55 °C IC_50_ < 15.6 µg/mL ≈90% cell death with 500 µg/mL (MTT)	Xenograft mice (U14 cells) n.r.	Minimal effects (body weight) Minimal tissue damage (histological)	λ = 980 nm P = 0.8 W/cm^2^ t = 15 min	―	ΔT ≈ 35 °C Tumor did not grow in 14 d and was 7× smaller than control (volume) Noticeable tumor tissue damage (histological)	[48]
GO/MnWO_4_/PEG	4T1 HUVEC	90% and 80% (4T1 and HUVEC cell) with 100 µg/mL (MTT)	λ = 808 nm P = 0.6 W/cm^2^ t = 10 min	DOX 5 µg/mL AL ≈ 55%	ΔT ≈ 55 °C IC_50_ < 50 µg/mL ≈90% cell death with 100 µg/mL (MTT)	Xenograft mice (U14 cells) 0.6 mg/mouse	Minimal effects (body weight) Minimal tissue damage (histological)	λ = 808 nm P = 0.6 W/cm^2^ t = 10 min	DOX 0.2 mg/mouse AL ≈ 55%	ΔT ≈ 27 °C Tumor abl. in 12 d. (volume) Noticeable tumor tissue damage (histological)	[5]
LOGr-Pc-LHRH	A2780/AD RBC	90% (A2780/AD) with 125 µg/mL (MTT) Minimal hemolytic activity (RBC) with 44 µg/mL	λ = 690 nm P = 0.95W/cm^2^ t = 15 min	Pc 4 µg/mL	ΔT ≈ 35 °C IC_50_ < 1.8 µg/mL ≈95% cell death with 7 µg/mL (Calcein AM)	Xenograft mice (A2780/AD cells) 1 mg/Kg	―	―	―	Only diagnostic by fluorescence tumor imaging	[81]
**Therapeutic outcomes and toxicity evaluation of graphene-based nanomaterials containing NGO**											
GO-DOX	―	―	―	―	―	Xenograft mice (H1975 cells) n.r.	Undetectable tissue damage (histological) Minimal effects (body weight)	―	DOX 8 mg/kg AL = 133%	Tumor reduced ≈75% in 14 d (volume)	[71]
ICG-FeCl_3_@GO	G361	―	λ = 785 nm P = 1 W/cm^2^ t = 20 min	ICG n.r.	IC_50_ n.r. 99% cell death [GBNs] n.r (MTT, Trypan blue)	―	―	―	―	―	[83]
GO@Ag-DOX-NGR	MCF-7	≈90% with 10 µg/mL (SRB)	λ = 808 nm P = 2 W/cm^2^ t = 3 min	DOX 4 µg/mL AL ≈ 82%	ΔT = 18 °C 0.5 < IC_50_ < 1 µg/mL ≈94% cell death with 4.88 µg/mL (SRB)	Xenograft mice (S180) 6.1 mg/Kg	Minimal effects (body weight) Minimal tissue damage (histological)	λ = 808 nm P = 2 W/cm^2^ t = 3 min	DOX 5 mg/kg AL ≈ 82%	Tumor did not grow in 13 d and was 5× smaller than control Survival≥ 14 d Noticeable tumor tissue damage (histological)	[76]
GO-PEG-DVDMS	PC9	≈70% with 3 µg/mL (MTT)	- PDT: λ = 630 nm P = 3 J/well t = n.r. - PTT: λ = 808 nm P = 1 W/cm^2^ t = 3 min	DVDMS n.r	IC_50_ ≈ 0.25 µg/mL 90% cell death with 3 µg/mL (MTT)	Xenograft mice (PC9 cells) 1 mg/Kg	Minimal effects (body weight)	- PDT: λ = 630 nm 50 J t = n.r. - PTT: λ = 808 nm P = 1 W/cm^2^ t = 10 min	DVDMS 2 mg/kg	ΔT = 25 °C Tumor abl. in 2 d (volume)	[88]
IO/GO-COOH	HeLa	95% with [Fe] = 200 µg/mL (MTT)	λ = 808 nm P = 2 W/cm^2^ t = 5 min	―	ΔT = 60 °C IC_50_ n.r. ≈100% cell death with 150 µg/mL (MTT)	Xenograft mice (S180 cells) 37.5 µg /mouse	Minimal effects (body weight)	λ = 808 nm P = 1 W/cm^2^ t = 5 min	―	ΔT > 37 °C Tumor abl. in 2 d (volume) Survival ≥ 60 d	[60]
GO-PEG-CysCOOH	4T1	≈100% with 250 µg/mL (MTT)	λ = 808 nm P = 0.5 W/cm^2^ t = 3 min	―	ΔT ≈ 25 °C IC_50_ n.r. >90% cell death with 250 µg/mL (MTT)	Xenograft mice (4T1) 450 μg/mouse	No detectable effects (body weight) Minimal tissue damage (histological)	λ = 808 nm P = 0.5 W/cm^2^ t = 5 min	―	ΔT ≈ 38 °C Tumor abl. in 2 d(volume) Survival ≥ 60 d	[73]
Au@NGO	HeLa cells	≈80% with 200 µg/mL (MTT)	―	DOX 25 µg/mL AL ≈ 2%	0.375 < IC_50_ = 1.25 µg/mL >90% cell death with 2 µg/mL (MTT)	―	―	―	―	―	[68]
NGO-PEG-FA	B16F0	≈90% with 75 µg/mL (MTT)	PTT λ = 808 nm P = 0.32 W/cm^2^ t = 15 min PTT+PDT λ = 980 nm P = 0.32 W/cm^2^ t = 18 min	―	- PTT ΔT ≈ 18 °C 50 < IC_50_ < 75 µg/mL 65% cell death with 75 µg/mL - PTT+PDT: ΔT ≈ 11 °C IC_50_ = 25 µg/mL 85% cell death with 75 µg/mL (MTT)	Xenograft mice (B16F10 cells) 8 mg/Kg	―	PTT λ = 808 n P = 0.25 W/cm t = 8 mi PTT+PDT λ = 980 nm P = 0.25 W/cm^2^ t = 10 min	―	- PTT: ΔT = 8.8 °C Tumor slow growth in 14 d Survival ≤ 32 d - PDT+PTT: ΔT = 1.8 °C Tumor abl. in 14 d (volume) Survival ≥ 40 d	[7]
NGO-IR-808	A549; Lewis lung	Negligible toxicity with 10 µM (CCK-8)	λ = 808 nm P = 2 W/cm^2^ t = 5 min	―	ΔT = 34 °C - A549 cells: IC_50_ = 5 µg/mL 95% cell death with 10 µM - Lewis lung cells: 2.5 < IC_50_ <5 µg/mL 100% cell death with 10 µM (CCK-8)	Xenograft mice (A549; Lewis lung cells) 10 mg/Kg	No detectable effects (body weight) Minimal tissue damage (histological)	λ = 808 nm P = 1 W/cm^2^ t = 5 min	―	ΔT = 23.5 °C Tumor abl. in 3–4 d. (volume) Noticeable tumor tissue damage (histological)	[67]
NGO-PEG-ICG/PTX	MG-63	≈100% with 200 µg/mL (w/o PTX) (CCK-8)	―	PTX 100 µg/mL AL ≈ 90%	IC_50_ = 20 µg/mL 90% cell death with 100 µg/mL of PTX (CCK-8)	Xenograft mice (MG-63) 10 mg/kg	No detectable effects (body weight) Minimal tissue damage (histological)	―	PTX 9 mg/kg AL ≈ 90%	Tumor abl. in 15 d (volume) Noticeable tumor tissue damage (histological)	[91]
NGO-UCNPs-Ce6	HeLa; L929	>95% with 800 µg/mL (MTT)	λ = 808 nm P = 0.72 W/cm^2^ t = 10 min	―	ΔT = 47 °C IC_50_ = 25 µg/mL 85% cell death with 800 µg/mL (MTT)	Xenograft mice (U14 cells) n.r.	No detectable effects (body weight) Minimal tissue damage (histological)	λ = 808 nm P = 0.72 W/cm^2^ t = 10 min	―	Tumor did not grow in 14 d and was 9× smaller than control (volume) Noticeable tumor tissue damage (histological)	[57]
UCNP@ NGO	4T1	>90% with 400 µg/mL (MTT)	λ = 808 nm P = 2 W/cm^2^ t = 10 min	―	ΔT = 47 °C IC_50_ = 100 µg/mL >90% cell death with 400 µg/mL (MTT, Cytometry)	Xenograft mice (4T1 cells) n.r.	Minimal effects (body weight) Minimal tissue damage (histological)	λ = 808 nm P = 1 W/cm^2^ t = 5 min	―	ΔT = 23 °C Tumor abl. in 6 d (volume)	[64]
**Therapeutic outcomes and toxicity evaluation of graphene-based nanomaterials containing rGO**											
BSA/nano-rGO	MCF-7 cells	100% with 0.04 µg/mL (MTT)	λ = 808 nm P = 6 W/cm^2^ t = 5 min	―	ΔT = 30 °C IC_50_ = 0.108 µg/mL 80% cell death with 0.15 µg/mL (MTT)	Xenograft mice (MCF-7) 20 mg/kg	Undetectable tissue damage (histological)	λ = 808 nm P = 0.6 W/cm^2^ t = 5 min	―	ΔT = 18 °C Noticeable tumor tissue damage (histological)	[74]
rGONM-PEG-Cy7-RGD	―	―	―	―	―	Xenograft mice (U87MG cells) 0.2 mg/mouse	Minimal effects (body weight)	λ = 808 nm P = 0.1 W/cm^2^ t = 7 min	―	Tumor abl. in 3 d (volume) Survival ≥ 90 d	[46]
rGO- Fe_2_O_3_@Au NPs	HeLa	≈90% with 50 µg/mL (MTT)	λ = 808 nm P = 2 W/cm^2^ t = 5 min	DOX n.r. AL≈100%	ΔT = 30 °C 10 < IC_50_ < 20 µg/mL 97% cell death with 50 µg/mL (MTT)	―	―	―	―	―	[50]
rGO nanosheets	KB	≈95% with 20 µg/mL (CCK-8)	λ = 808 nm P = 1.2 W/cm^2^ t = 3 min	―	ΔT = 51 °C IC_50_ n.r. >90% cell death with 20 µg/mL (CCK-8)	Xenograft and orthotopic mice (KB cells) 5 mg/kg	Minimal effects (body weight)	λ = 808 nm P = 1.2 W/cm^2^ t = 3 min	―	ΔT = 13 °C Tumor abl. in 3 d (volume) highest % of apoptotic cells (histological) Survival ≥ 50 d	[69]
^131^I-RGO-PEG	4T1	≈90% with 200 µg/mL (CCK-8)	λ = 808 nm P = 0.5 W/cm^2^ t = 10 min	^131^I 100 µCi	ΔT = 51 °C IC_50_ n.r. 90% cell death with 100 µg/mL (CCK-8)	Xenograft and orthotopic mice (4T1cells) 10 mg/kg	Minimal effects (body weight)	λ = 808 nm P = 0.2 W/cm^2^ T = 20 min	^131^I 200 µCi/ mouse	ΔT ≈ 18 °C Tumor abl. in 16 d (volume) Noticeable tumor tissue damage (histological)	[51]
rGO-AuNRVe	U87MG	100% with 2.4 nM (CCK-8)	λ = 808 nm P = 0.25 W/cm^2^ t = 5 min	DOX 6.4 µg/mL AL ≈ 65%	IC_50_ = 0.63 µg/mL (DOX) 90% cell death with 6.4 µg/mL of DOX (CCK-8)	Xenograft and orthotopic mice (U87MG) 10 mg/kg	Minimal effects (body weight) Minimal tissue damage (histological)	λ = 808 nm P = 0.25 W/cm^2^ t = 5 min	DOX AL ≈ 65%	ΔT = 13 °C Tumor abl. in 14 d (volume) Survival≥ 40 d	[79]
anti-EGFR-PEG-rGO@ CPSS-Au-R6G	A549	100% with 100 µg/mL (MTT)	λ = 808 nm P = 0.5 W/cm^2^ t = 5 min	―	IC_50_ n.r. 72% cell death with 100 µg/mL (MTT)	―	―	―	―	―	[6]
ICG-PDA-rGO	4T1	≈90% with 20 µg/mL (MTT)	λ = 808 nm P = 0.6 W/cm^2^ t = 5 min	―	ΔT = 30 °C IC_50_ = 10 µg/mL 85% cell death with 40 µg/mL (MTT)	Xenograft and orthotopic mice (4T1 cells) 200 µg/mouse	Minimal effects (body weight)	λ = 808 nm P = 0.6 W/cm^2^ t = 5 min	―	Tumor abl. in 3 d (volume) Survival ≥ 40 d	[59]
rGO-GSPs	U87MG	≈100% with 100 µg/mL (MTT)	λ = 808 nm P = 0.8 W/cm^2^ t = 5 min	―	ΔT = 40 °C IC_50_ n.r. 85% cell death with 100 µg/mL (MTT)	Xenograft mice (U87MG cells) 200 μg/mouse	No detectable tissue damage (histological)	λ = 808 nm P = 0.8 W/cm^2^ t = 5 min	―	ΔT = 28 °C Tumor abl. in 2 d (volume) Noticeable tumor tissue damage (histological) Survival ≥ 40 d	[66]
rGO-mfHSA	HepG2	≈100% with 20 μg/mL (CCK-8)	λ = 808 nm P = 2 W/cm^2^ t = 5 min	―	ΔT ≈ 30 °C IC_50_ = 10 μg/mL >90% cell death (CCK-8)	Xenograft mice (HepG2) 200 μg/mouse	No detectable effects (body weight)	λ = 808 nm P = 1 W/cm^2^ t = 10 min	―	ΔT ≈ 20 °C Tumor growth. reduced 4× in 20 d (volume)	[93]
FA-PEG-Lip@rGO/ Res	A549 MCF-7	>90% with 80 µg/mL (w/o Res) (MTT)	λ = 780 nm P = 0.6 W/cm^2^ t = 10 min	Res 56 µg/mL AL ≈ 70%	IC_50_ ≈ 10 µg/mL >98% cell death with 80 µg/mL (MTT)	Xenograft mice (MCF-7 cells) 2.2 mg/kg	No detectable effects (body weight)	λ = 780 nm P = 0.6 W/cm^2^ t = 5 min	Res AL ≈ 70%	ΔT = 17 °C Tumor abl. in 10 d (volume)	[58]
ArGO	―	―	―	―	―	Xenograft mice (SCC7 cells) 5 mg/kg	100% survival one day after i.v.; normal blood test results (50 mg/kg)	λ = 808 nm P = 1.5 W/cm^2^ t = 3 min	―	ΔT = 16 °C Tumor abl. in 10 d (volume) Survival≥ 50 d	[77]
AAP10-pDA/rGO	MCF-7	≈100% with 160 µg/mL (MTT)	λ = 808 nm P = 1.5 W/cm^2^ t = 5 min	AAP10 50 nM AL ≈ 0.024%	ΔT = 18 °C IC_50_ n.r. ≈80% cell death with 120 µg/mL (MTT)	Xenograft mice (4T1 cells) 0.3 mg/mouse	Minimal effects (body weight) No detectable tissue damage (histological)	λ = 808 nm P = 1.5 W/cm^2^ t = 5 min	AAP10 AL ≈ 0.024%	ΔT = 25 °C Tumor abl. in 7 d (volume) Noticeable tumor tissue damage (histological)	[90]
**Therapeutic outcomes and toxicity evaluation of graphene-based nanomaterials containing GQDs**											
cGdots	MDA-MB231	>70% with 500 µg/mL (MTT)	λ = 670 nm P = 0.3 W/cm^2^ t = 30 min	―	ΔT ≈ 25 °C IC_50_ ≤ 50 µg/mL >70% cell death with 500 µg/mL (MTT)	Xenograft mice (MDA-MB231 cells) 75 µg/mouse	No detectable effects (body weight)	λ = 670 nm P = 0.3 W/cm^2^ t = 30 min irr. every other day	―	ΔT = 17 °C Tumor growth reduced 2× in 14 d (volume) Noticeable tumor tissue damage (histological)	[45]
GQDs	HeLa	>90% with 1.8 µM (MTT)	λ = 670 nm P = 6.5 mW/cm^2^ t = 10 min	PpIX n.r.	0.036 < IC_50_ < 0.09 µM >80% cell death with 1.8 µM (MTT)	Xenograft mice (MDA-MB231 cells) 80 µg/mouse	No detectable effects (body weight)	λ = 400–800 nm P = 80 mW/cm^2^ t = 10 min irr. day 1 and 7	PpIX n.r.	Tumor abl. in 20 d (volume)	[44]
PLA-PEG-grafted GQDs (f-GQDs)	HeLa	≈90% cell viability with 140 µg/mL (MTT)	―	IP and ASODN 50 nM	IC_50_ n.r. 32% cell death with 14 µg/mL (Cytometry)	―	―	―	―	―	[55]
AS1411@ GQD	A549	100% cell viability with 5 µM (MTT)	λ = 808 nm P = 2 W/cm^2^ t = 10 min	AS1411n.r.	ΔT ≈ 13 °C (in H_2_O) 50% cell death with 5 µM (MTT)	―	―	―	―	―	[84]
HA-GQD -SiO_2_ NPs	HeLa	100% with 4 µM Hypo-crellin (MTT)	λ = 470 nm	Hypocre-llin 4 µM	IC_50_ n.r. 80% cell death with 4 µM Hypocrellin (MTT)	―	―	―	―	―	[94]
GQDs@Cys-BHC	L929 HeLa MDA-MB-231	Low toxicity with 200 µg/mL (w/o BHC) (Trypan blue, MTT)	―	BHC ≈0.4 mM AL ≈ 88%	IC_50_ = 200 µg/mL 40–50% cell death with 200 µg/mL (Trypan blue, MTT)	―	―	―	―	―	[82]
Fe_3_O_4_@SiO_2_ @GQDs-FA/DOX	HeLa	>90% with 50 µg/mL (MTT)	λ = 808 nm P = 0.3 W/cm^2^ t = 10 min	―	IC_50_ = 1 µg/mL 85% cell death with 50 µg/mL (MTT)	―	―	―	―	―	[80]
GQD-MSN- -DOX	4T1	≈95% with 100 μg/mL (CCK-8)	λ = 808 nm P = 2.5 W/cm^2^ t = 3 min	DOX 4.5 μg/mL AL ≈ 4.8%	ΔT ≈ 20 °C IC_50_ n.r. ≈90% cell death with 100 µg/mL (CCK-8)	―	―	―	―	―	[89]
GQD-PEG-P	A549 MCF-7	100% with 100 µg/mL (MTT)	λ = 980 + 636 nm P = 0.72 W/cm^2^ t = 10 min	―	ΔT = 30 °C IC_50_ n.r. 90% cell death with 100 µg/mL (MTT)	―	―	―	―	―	[49]
DOX@GQD- -P-Cy	4T1	≈95% with 4 µg/mL (w/o DOX) (MTT)	―	DOX 3.3 µg/mL AL ≈ 82.5%	IC_50_ = 1 µg/mL ≈98% cell death with 4 µg/mL (MTT)	Xenograft mice (4T1 cells) 1 µg /mouse	Minimal effects (body weight)	―	DOX 0.8 µg /mouse AL ≈ 82.5%	Tumor growth decreased 4× (volume) Noticeable tumor tissue damage (histological) Survival ≤ 20 d	[54]
DL-GQD-comp	BT-474	≈90% with 100 µg/mL (w/o DOX) (CCK-8)	―	DOX 8.8 µM AL ≈ 5.3%	50 < IC_50_ < 100 µg/mL ≈80% cell death with 100 µg/mL (CCK-8)	―	―	―	―	―	[63]
IR780/GQD-FA	HeLa	≈90% with 30 µg/mL (CCK-8)	λ = 808 nm P = 1 W/cm^2^ t = 5 min	―	ΔT = 28 °C IC_50_ = 10 µg/mL ≈98% cell death with 30 µg/mL (CCK-8)	Xenograft mice (HeLa cells) 2 mg/kg	No detectable effects (body weight)	λ = 808 nm P = 1 W/cm^2^ t = 5 min	―	ΔT = 23 °C Tumor abl. in 6 d (volume) Survival ≥ 60 d	[65]
SCNA (DOX/GQD)	RG2	≈100% with 10 µg/mL (w/o DOX) (alamar blue)	λ = 808 nm P = 2 W/cm^2^ t = 5 min	DOX 2 µg/mL AL n.r.	IC_50_ n.r. ≈75% cell death with 10 µg/mL (alamar blue)	Xenograft mice (RG2 cells) 0.2 mg/mouse	No detectable effects (body weight)	λ = 808 nm P = 2 W/cm^2^ t = 10 min	DOX 2 µg/mL AL n.r	ΔT = 10 °C Noticeable tumor tissue damage (histological)	[8]

Table 3 abbreviations: GBNs—Graphene-based nanomaterials; GO—Graphene oxide; NGO—Nanographene oxide; rGO—Reduced Graphene oxide; GQDs—Graphene Quantum Dots; AL (wt%)—Active’s loading; d–day(s); n.r.—not reported; abl.—completely ablated; w/o—without; NIR laser– Near infrared laser; (λ, P, and t)—characteristics of the laser: wavelength; power and time; ΔT—temperature increase; IC_50_—concentration of GBNs required to kill 50% of cells; PTT—photothermal therapy; PDT—photodynamic therapy; AAP10—Antiarrhythmic peptide 10 (promotes bystander effect); Abs—integrin α_v_β_3_ mAb (targeting ligand); Ag—silver; AGE-aptamer—targets melanoma inhibitor of apoptosis protein (ML-IAP) overexpressed in melanoma cells; anti-EGFR—anti-epidermal growth factor receptor (targeting ligand); APGA—amphiphilic poly-γ-glutamic acid; ArGO—rGO coated with amphiphilic poly-γ-glutamic acid; AS1411—aptamer of 26-base guanine-rich short oligonucleotide (targeting ligand); ASODN—survivin antisense oligodeoxynucleotide; Au—gold; AuNPs—gold nanoparticles; AuNRVe—gold nanorod vesicles; AuNR—Gold nanorods; AuNS—Gold nanostars; Bi_2_Se_3_—Bismuth Selenide; BHC—Berberine hydrochloride; BPEI—Branched polyethylenimine; BSA—bovine serum albumin; Ce6—Chlorin e6 (photosensitizer); cGdots—carboxylated graphene dots; Cy5.5—Cyanine 5.5 (NIR dye and photosensitizer); Cy7—Cyanine 7 (NIR dye and photosensitizer); Cys—Cysteamine hydrochloride (NIR dye); Cys-COOH—Cysteine- rich Carboxy-terminal domain CPGA—theranostic probe formed by Cy5.5 (NIR dye) labelled-matrix metalloproteinase-14 (MMP-14) substrate (CP) conjugated onto the GO/Au complex (GA); CPSS—carbon porous silica nanosheets; Cur—curcumin; DL-GQD-comp—doxorubicin hydrochloride loaded GQD complex; DOX—doxorubicin hydrochloride; DSPE—1,2-distearoyl-*sn*-glycero-3-phosphoethanolamine; DVDMS—bis[1-[6,7-bis[2-(sodium carbonate ethyl]-1,3,5,8,-tetramethyl-2-vinyl-porphin-4-yl]ethyl]ether (photosensitizer); FA—Folic acid (target ligand); FeCl_3_—Iron chloride; Fe_2_O_3_ and Fe_3_O_4_—Iron oxide nanoparticles; Gd—Gadolinium; GDH—Graphene–DOX conjugate in HA nanogel; GSPs—gold superparticles; HA—hyaluronic acid (target ligand); HA-GQD—complex of Hypocrellin A (photosensitizer), HA and GQD; HER—Herceptin, monoclonal antibody that targets HER2 Positive Metastatic Breast Cancer (target ligand); HTPGS—N-acetyl histidine-functionalized D-α-tocopherol polyethylene glycol 1000 succinate; ^131^I—Iodine-131 (radioisotope); ICG—NIR fluorescence dye; IO—iron oxide; IR780—IR780 iodide (NIR dye); IR-808—Heptamethine indocyanine dye (photosensitizer); LHRH—luteinizing hormone-releasing hormone peptide; Lip—Phospholipids; LOGr—low-oxygen graphene; mfHSA—multifunctional human serum albumin—HSA functionalized with indocyanine green (ICG) and lactobionic acid (LA); MMP-14(P)—Peptide substrate of MMP-14, a key endopeptidase that is overexpressed on tumor cell surface; MnWO_4_—manganese tungstate; MSN—mesoporous silica nanoparticles; NGR—Asn-Gly-Arg peptide that can selectively recognize CD13 isoform selectively overexpressed in tumor vasculature and certain tumor cells (target ligand); OA—Oleic acid; P—porphyrin; PAH—poly (allylamine hydrochloride); Pc—phthalocyanine; P-Cy—Cyanine 5.5 dye conjugated to GQD though a cathepsin D-responsive peptide (P); PDA or pDA—Polydopamine (reduces GO improves water solubility and biocompatibility and increases NIR absorption); PEG—Polyethylene glycol; PheoA—Pheophorbide A (photosensitizer); PLA—polylactic acid; PpIX—protoporphyrin IX (photosensitizer); PTX—Paclitaxel; PVP—polyvinylpyrrolidone; R6G—Rhodamine 6G; Res—Resveratrol; rGONM—reduced graphene oxide nanomesh; RGD—arginine–glycine–aspartic acid-based peptide (target ligand); SCNA—size-changeable graphene quantum dot nanoaircraft; SiO_2_ NPs—silicon dioxide nanoparticles; UCNPs—upconversion luminescence nanoparticles; ZnFe_2_O_4_—Zinc ferrite nanoparticles; ZnPc—Zinc phthalocyanine (photosensitizer); ZnS:Mn—manganese-doped zinc sulfide nanoparticles. Methods: Alamar Blue—cell proliferation assay designed to measure cell proliferation and cytotoxicity in various cell lines; Calcein AM—non-fluorescent, hydrophobic compound that easily permeates intact live cells; it can be used in a cell viability assay once, by hydrolysis, converts in calcein, a hydrophilic and strongly fluorescent compound; CCK-8—Cell Counting Kit-8 that allows sensitive colorimetric assays for the determination of cell viability in cell proliferation and cytotoxicity assays; MTT—colorimetric assay for assessing cell metabolic activity based on conversion of 3-(4,5-dimethylthiazol-2-yl)-2,5-diphenyltetrazolium bromide to formazan; SRB –Sulforhodamine B assay used for cell density determination, based on the measurement of cellular protein content; Trypan Blue—dye exclusion test, used to determine the number of viable cells present in a cell suspension. Cells: 4T1—mouse breast carcinoma cell line; A2780/AD—multidrug resistant human ovarian carcinoma cell line; A549—lung cancer cell line; B16F0—mouse melanoma cell line; B16F10—murine melanoma cell line; BT474—human breast cancer cell line; CHO-K1—Chinese Hamster Ovary cell line; EMT6—mouse breast cancer cell line; G361—human malignant melanoma cancer cells; H1975—lung adenocarcinoma cells; HeLa- human cervical cancer cells; HCT166—human colon cancer cells; HepG2—human hepatocellular carcinoma cell line; HT1080—human fibro-sarcoma cells; HUVEC - Human umbilical vein endothelial cells; KB—Human KB epidermal carcinoma cells; L929—mouse fibroblasts; Lewis lung—mouse lung carcinoma cells; MCF-7—human breast adenocarcinoma cell line; MCF-10—normal breast cells; MDA-MB-231—breast cancer cells; MDCK—Canine Cocker Spaniel Kidney non-cancerous cell line; MG-63—human osteosarcoma cell line; RBC—red blood cells; PC9—human adenocarcinoma cell line; RG2—mouse glioblastoma cell line; S180—murine sarcoma cancer cell line; SCC7—mouse squamous cell carcinoma cell line; SKBR-3—human epithelial breast cancer cells; U87MG—human glioblastoma astrocytoma cells; U14—mouse uterine cervical carcinoma cells; U87MG—human glioblastoma astrocytoma cells.

**Table 4:** List of targeting moieties used to functionalize GBNs and direct them to cancer cells.

Ligands	Function	Ref.
Abs	Targets integrin (α_v_β_3_) receptor overexpressed in cancer cells	[72]
AGE-aptamer	Targets melanoma inhibitor of apoptosis protein (ML-IAP) overexpressed in melanoma cells	[83]
Anti-EGFR	Targets the epidermal growth factor receptor (EGFR) of lung cancer cells	[6]
AS1411	Aptamer specific to malignant melanoma	[84]
BPEI	Targets the organic anion transporting polypeptides (OATPs) overexpressed in cancer cells	[46]
FA	Targets folic acid receptors overexpressed in cancer cells	[7,47,58,65,75,80,86]
HA	Targets CD44 receptors, a cell surface adhesion receptor that is highly expressed in many cancers and regulates metastasis	[62]
HER	Targets HER2+ receptors in breast cancer cells	[63]
HSA-LA	Generates galactose residues that targets asialoglycoprotein receptor (ASGP-R), highly expressed on the surface of hepatocellular carcinoma cells (HCC)	[93]
MMP-14(P)	Targets the overexpressed endoperoxidase in tumor cell membrane	[56]
NGR	Targets CD13 isoform selectively overexpressed in tumor vasculature and certain tumor cells	[76]
RGD	Targets integrin α_v_β_3_ mAb overexpressed in cancer cells	[46]

Table abbreviations: Abs—integrin αvβ3 mAb; anti-EGFR—anti-epidermal growth factor receptor; AS1411—aptamer, 26-base guanine-rich short oligonucleotide; BPEI—Branched polyethylenimine, cationic polymer that can be used to condense nucleic acids in gene therapy or as targeting agent; FA—Folic acid; HA—hyaluronic acid; HER—Herceptin, monoclonal antibody; HSA-LA—human serum albumin functionalized with lactobionic acid (LA); MMP-14(P)—metalloproteinase-14 substrate; NGR—Asn-Gly-Arg peptide; RGD—arginine–glycine–aspartic acid-based peptide.

## Supplementary Materials

The following are available online at http://www.mdpi.com/1999-4923/10/4/282/s1. Table S1: Therapeutic strategies of theranostic nanosystems. Table S2: Optical diagnostic strategies of theranostic nanosystems. Table S3: Non-optical diagnostic strategies of theranostic nanosystems. Table S4: PRISMA (Preferred Reporting Items for Systematic review and Meta-Analysis) checklist: recommended items to address in a systematic review. Table S5: Checklist for assessing the quality of the studies. Table S6: Biodistribution information and therapeutic outcomes obtained with rGO formulations. Table S7: Biodistribution information and therapeutic outcomes obtained with GO, NGO, and GQDs based formulations.
