# Supplementary material for: A Systematic Review and Critical Analysis of the Role of Graphene-Based Nanomaterials in Cancer Theranostics
Source: Pharmaceutics. 2018 Dec 16;10(4):282. doi: 10.3390/pharmaceutics10040282 (PMC6321636; doi:10.3390/pharmaceutics10040282)
Supplement: Supplementary file 1 [file pharmaceutics-10-00282-s001.pdf]

pharmaceutics

MDPI

Supplementary Materials: A Systematic Review and Critical Analysis of the Role of Graphene-Based Nanomaterials in Cancer Theranostics

Teresa Viseu, Carla M. Lopes, Eduarda Fernandes, Maria Elisabete C.D. Real Oliveira, and Marlene Lúcio

Table S1. Therapeutic strategies of theranostic nanosystems.

Therapeutic Strategies	Agent	Mechanism	Advantages	Disadvantages	REF
Chemotherapy	Anticancer drugs loaded in several types of nanocarriers	Can cause damage or stress cells by several mechanisms (e.g. interference with angiogenesis and interference with cellular division) which may then lead to cell death if apoptosis is initiated.	There is an extensive knowledge of chemotherapeutical arsenal as it is the base of classical therapy of cancer;<br>Toxic effects are reduced by incorporation in the nanocarriers;<br>Nanocarriers benefit from EPR effect and tend to locate at tumor sites.	Toxic effects are reduced, but are still observed due to nanocarriers distribution in off-target tissues;<br>Tumor resistance and recurrence;<br>Tumor distribution by EPR is observable in vitro, but not always translatable to in vivo. Targeting moieties and other strategies are necessary.	[1]
Genetic therapy	Genes, gene segments, or oligonucleotides loaded in several types of nanocarriers (e.g. cationic lipid and polymeric nanocarriers)	Deactivation of oncogenes;<br>Replacement of non-functioning tumor suppressor genes;<br>Cell death or repair of normal cellular functions;<br>Protection of normal cells from drug-induced toxicity or activation of immune cells for cancer cells destruction.	Incorporation of genes, gene segments or oligonucleotides in nanocarriers offers protection against enzyme-induced degradation and/or inactivation.	Three key factors are necessary for the success of nanocarriers in gene therapy: high loading efficiency, capacity for being delivered from endosomes to cytosol and capacity to release the genetic content. Often nanocarriers fail some of these key steps and may not reach high levels of transfection;<br>Cytotoxicity and biocompatibility issues are observed due to high density of positive charges in the nanocarriers.	[2]
Photothermal Therapy (PTT)	PT agents, such as: AuNs; CBNs; CuS NPs; Pd NS; PB NPs	An optical-absorbing agent converts NIR light into thermal energy and the local temperature increases (hyperthermia);<br>Mild hyperthermia (43-50°C) causes increased membrane permeability, dysfunctional membrane transport, metabolic signaling disruption that leads to cell apoptosis;<br>Strong hyperthermia (>50°C) causes necrotic cell death due to disruption of cellular membrane and protein denaturation.	Low cost;<br>Non-invasive and remote-control alternative to classical therapy;<br>Localized and specific tumor treatment;<br>Cellular internalization of the nanocarriers is not required;<br>Great penetration depth in biological tissues.	PTT as a single therapy is generally not enough for complete tumor ablation;<br>Despite the tissue penetration, the heat distribution within tumor is heterogeneous and laser intensity decays with tissues depth;<br>Usage of lasers with high power density can harm healthy tissues.	[3]

Photodynamic Therapy (PDT)	PS agents (e.g. porphyrin derivates) loaded in several types of nanocarriers	A PS agent is excited to a singlet state by photon absorption;<br>The excited single state decays to a lower-energy excited triplet state through intersystem crossing;<br>In the excited triplet state, PS transfers an electron to: (i) different molecules producing ROS: $O_2^{\bullet -}$, H_2O_2, $HO^{\bullet}$or (ii) oxygen originating $^1O_2$;<br>ROS interact with cellular components (lipids, proteins, nucleic acids) causing oxidative stress and cell death.	Non-invasive and target specific;<br>Reduced side effects, causing less injury to healthy tissues and minimal systemic toxicity;<br>Rapid healing process after therapy;<br>Repeated doses can be administrated without exceeding total dose;<br>Avoids MDR.	For efficient generation of $^1O_2$it is required the presence of 3 components: PS, light and oxygen. Though, tumor microenvironments are mostly hypoxic causing a low production of $^1O_2$ and a limited therapeutic efficiency;<br>Clinical use of most PS is limited due to prolonged cutaneous photosensitivity, poor water solubility, poor photostability and incapacity to be activated by NIR light, which causes poor tissue penetration and restricts PDT to localized and superficial tumors.	[4,5]
Magnetic Hyperthermia (MHT)	MNPs made of transition metals, such as: Fe; Ni; Co; Mn and its oxides. Examples:<br>Fe_3O_4, γ-Fe_2O_3 or $M_xFe_{3-x}O_4$<br>(M = divalent metallic cations)	The MNPs exposed to an external alternating magnetic field can convert magnetic energy in thermal energy by Néel or Brownian relaxation mechanisms;<br>When the application of the magnetic field is quicker than the MNPs relaxation time, the delay in the relaxation of the magnetic moments will cause heat generation (magnetic hyperthermia);<br>Magnetic hyperthermia (42-45°C) causes enhanced membrane permeability, dysfunctional membrane transport, metabolic signaling disruption that leads to cell death by apoptosis.	Non-invasive (by i.v. administration) and remote-control alternative to conventional tissues;<br>Highly localized and specific tumor treatment. Its selectivity is due to the natural transparency of the human body to the magnetic field;<br>Cellular internalization of the MNPs is not required.	The external field parameters must be optimized to maximize the hyperthermia effect in cancer cells, while preserving healthy cells and assuring patient comfort during treatment;<br>The electric permittivity of biological tissues is sufficiently high to originate unwanted currents, which can provoke non-selective hyperthermia and unmanageable "hot spots";<br>i.v. administration results in a low MNPs accumulation rate in the tumor;<br>Intratumor administration assures MNPs accumulation in tumor, but it is invasive;<br>MNPs tend to accumulate in the liver and could cause liver damage.	[6]

Table S2. Optical diagnostic strategies of theranostic nanosystems.

Optical Imaging Strategies	Agent	Mechanism	Advantages	Disadvantages	REF
Fluorescence imaging (FI)	QDs, CDs, GQDs, NPs loaded with fluorescent dyes	- Emission of low-energy fluorescence when a fluorophore is excited by a high-energy light (usually UV or visible light)	- Highly sensitive and high temporal resolution<br>- Cost-effectiveness and easy handling;<br>- Multiplexed biological labeling and imaging.	- Limited depth penetration;<br>- Auto-fluorescence from biological tissues;<br>- Photobleaching.	[7–9]
Two Photon Fluorescence Imaging (2PFI) or Upconversion Luminescence Imaging (UCLI)	GQDs, UCNPs, such as: $NaYF_4:Yb^{3+}$, Er^{3+} or $NaYF_4$: Yb^{3+}, Tm^{3+}	- It is an anti-Stokes type emission where two or more low-energy photons from NIR light are absorbed to produce higher energy emission in the visible region;	- Negligible photobleaching even after continuous exposure to high excitation energy levels;<br>- The use of NIR light as excitation reduces the biological tissues autofluorescence and increases penetration depths also reducing the photodamage of healthy tissues.	- UCNPs are normally not water soluble;<br>- Although surface modification can enhance their water solubility and biocompatibility, the procedures are time-consuming and may affect the luminescence efficiency.	[10,11]
Infrared Thermal Imaging (IR-TI) or Photothermal imaging (PTI)	PT agents, such as: AuNs; CBNs; CuS NPs; Pd NS; PB NPs	- It is based on the change in thermal state due to the absorption of radiation. Light absorbed and not lost by emission results in heating that can be recorded as an image.	- Direct: does not require any dye;<br>- Non-invasive;<br>- Safe;<br>- Provides real-time imaging	- Accurate temperature measurements can be difficult by wide emissivity and reflections derived from tissue surroundings.	[12,13]
Raman Spectroscopy and Surface Enhanced Raman Spectroscopy (SERS)	AuNPs; AgNPs, Raman-active dyes, such as Cy3, CBNs.	- A visible or NIR light interacts with the material originating inelastic scattering of photons that display a shift in frequency. The shift in energy gives information about the vibrational modes in the system;<br>- SERS: when molecules are adsorbed or located near a metallic nanostructure, enhancement of the Raman scattering occurs due to the resonant interaction of light with the surface plasmons excited at the surface of the structure.	- Raman spectroscopy can measure both morphological and chemical information in samples;<br>- Non-invasive, cost-effective and can achieve high chemical specificity based entirely on intrinsic molecular contrast in biological samples;<br>- Provides quantitative molecular information that can be translated into an objective diagnosis.	- The relatively low speed of Raman spectroscopy has been a main weakness for clinical translation;<br>- Raman scattering-based techniques are mostly performed on accessible tissue surfaces, for example, on the skin, in gastrointestinal tract, or intraoperatively.	[14,15]

Table S3. Non-optical diagnostic strategies of theranostic nanosystems.

Non-Optical Imaging Strategies	Agent	Mechanism	Advantages	Disadvantages	REF
Ultrasound Imaging (UI)	Nanobubbles; SiNPs; CNTs	- Based on the conversion of electrical signals into ultrasound waves that enter the body and travel through biological tissues;<br>- Some sound waves are reflected to a transducer and converted to electrical signals that are treated and presented as an image.	- Cost-effectiveness;<br>- Non-invasive;<br>- Provides real-time imaging;<br>- Safe (non-ionizing) and available in portable devices;<br>- Good temporal resolution;<br>- Provides quantification.	- Depth penetration of ultrasound waves is relatively weak;<br>- The imaging of hard tissues (e.g bones) and air is difficult by ultrasound due to their tendency to transmit sound waves. Thus, it is difficult to obtain ultrasound imaging of brain and stomach;<br>- Imaging quality is operator-dependent.	[7–9]
Photoacustic Imaging (PAI)	Materials with strong NIR absorption: Metal NPs such as AuNPs and PB NPs; SWCNTs; Organic dyes; Conducting polymeric NPs; CBNs	- When tissues are irradiated with nonionizing short laser pulses, the endogenous proteins absorb the energy and generate heat that leads to thermoelastic expansion;<br>- During contraction there is an emission of mechanical pressure waves at ultrasonic frequencies;<br>- The periodic sound waves can be detected by ultrasonic transducers that form an image by mapping the initial absorbed energy distribution	- Cost-effectiveness;<br>- Non- invasive;<br>- Safe (non-ionizing)<br>- Deep tissue/organ imaging;<br>- Good temporal resolution.	- The imaging of hard tissues (e.g bones) and air is difficult;<br>- Typically requires a contrasting agent with strong NIR absorbance to further improve imaging performance	[7,8,16]
Positron emission tomography (PET)	Radionuclides which emit positrons such as: ^{64}Cu; ^{18}F; ^{68}Ga; ^{124}I and radionuclide labelled CDs	- Nuclear medicine imaging;<br>- A cyclotron is used to generate short-lived or ultra-short-lived radionuclides that decay via positron emission producing photons 10-fold more energetic than X-rays.	- Excellent sensitivity;<br>- Quantification capabilities;<br>- Unlimited depth of penetration<br>- Better spatial resolution than SPECT.	- Lack of an anatomical reference frame and safety profile due to ionizing radiations;<br>- Risk of radiation exposure;<br>- Requires specialized and expensive equipment;<br>- Requires radionuclide facilities.	[7–9]
Single Photon Emission Computed Tomography (SPECT)	Radionuclides which emit γ rays such as: ^{99m}Tc; ^{123}I; ^{111}In; ^{67}Ga; and radionuclide labelled NPs	- Like PET mechanism, except it uses different nuclides which decay with the emission of single γ rays with different energies.	- Multiplexing capabilities: potential to detect multiple radionuclides simultaneously in contrast with PET;<br>- Unlimited depth of penetration;<br>- Lower cost and wider availability than PET.	- Lower sensitivity than PET;<br>- Lower spatial resolution than PET;<br>- Does not provide quantitative data;<br>- Lack of an anatomical reference frame and safety profile due to ionizing radiations;<br>- Risk of radiation exposure;<br>- Requires specialized and expensive equipment;<br>- Requires radionuclide facilities.	[7–9]

Computed Tomography (CT)	$NaLuF_4$/ $NaYbF_4$; Bi_2S_3; FePt; WO_x TaO_x; Iodinated NPs and AuNPs	- Relies on differential levels of X-ray attenuation by tissues within the body to produce three dimensional high-contrast anatomic images enabling delineation between various structures.	- Best clinical spatial resolution and good temporal resolution;<br>- Unlimited depth of penetration;<br>- Lower cost and wider availability than PET/SPECT.	- Poor sensitivity: requires high quantity of imaging agents;<br>- Lower spatial resolution than PET;<br>- Does not provide quantitative data;<br>- Safety issues due to the risks associated with the exposure to X-ray radiations and to the toxicity of ionizing radiations;<br>- Limited soft tissue visualization.	[7–9]
Magnetic Resonance Imaging (MRI)	Paramagnetic metals loaded in NPs or nanomaterials, such as: Iron oxide NPs (USPIO, SPION); Gd based NPs and Mn based NPs	- Consists in the application of radiofrequency pulses and it is based on the interaction between water protons and the applied magnetic field, with the image being formed through the absorption and emission pattern of the electromagnetic wave;<br>- To enhance the visibility of internal structures, contrast agents are used to shorten the relaxation times (t1 and t2) of water protons within body tissues.	- Good spatial resolution;<br>- Unlimited depth of penetration;<br>- Good soft tissue contrast;<br>- Provides both anatomical and functional information.	- Poor sensitivity: requires high quantity of imaging agents and demands long acquisition time;<br>- Does not provide direct quantitative data;<br>- No threat for any radiation exposure, but toxicity can result from the amount of contrast agents used;<br>- Requires expensive equipment	[7–9]

Tables S1, S2 and S3 abbreviations: AgNPs—Silver nanoparticles; AuNPs—Gold nanoparticles; AuNs—Gold nanomaterials; CBNs—Carbon based nanomaterials; CDs—Carbon dots; CNTs—Carbon nanotubes; CuS NPs—Copper sulfide nanoparticles; Cy3—Cyanine 3 dye; EPR—Enhanced permeability retention effect; Fe_3O_4–Spinel ferrite magnetite; γ-Fe_2O_3—Maghemite; GQD—Graphene quantum dots; H_2O_2—Hydrogen peroxide; HO^*—Hydroxyl radical; MDR—Multi-drug resistance; MNPs—Magnetic nanoparticles; $M_xFe_{3-x}O_4$–Mixed ferrites; NIR—Near infrared; NPs—Nanoparticles; O_2^{*-}—Superoxide anion; $^1O_2$– Reactive singlet oxygen; PB NPs—Prussian blue nanoparticles; Pd NS—Palladium nanosheets; PS agents—Photosensitive agents; PT agents—Photothermal agents; QDs—Quantum dots; SiNPs—Silica nanoparticles; SPION—Superparamagnetic iron oxide nanoparticles; SWCNTs—Single wall carbon nanotubes; TMDCs—two-dimensional (2D) transition metal dichalcogenides; UCNPs—Upconversion luminescence nanoparticles; USPIO—Ultrasmall superparamagnetic iron oxide; UV—ultraviolet.

Table S4. PRISMA (Preferred Reporting Items for Systematic review and Meta-Analysis) checklist: recommended items to address in a systematic review *.

Section and topic	Item No	Checklist item	Reported on Page
**TITLE**			
Title:	1	Identify the report as a systematic review, meta-analysis, or both	1
**ABSTRACT**			
Structured summary:	2	Provide a structured summary including, as applicable: background; objectives; data sources; study eligibility criteria, participants, and interventions; study appraisal and synthesis methods; results; limitations; conclusions and implications of key findings; systematic review registration number.	1
**INTRODUCTION**			
Rationale	3	Describe the rationale for the review in the context of what is already known	4
Objectives	4	Provide an explicit statement of the question(s) the review will address with reference to participants, interventions, comparators, and outcomes (PICO)	4
**METHODS**			
Protocol and registration	5	Indicate if a review protocol exists, if and where it can be accessed (e.g., Web address), and, if available, provide registration information including registration number.	-
Eligibility criteria	6	Specify study characteristics (e.g., PICOS, length of follow-up) and report characteristics (e.g., years considered, language, publication status) used as criteria for eligibility, giving rationale.	4
Information sources	7	Describe all information sources (e.g., databases with dates of coverage, contact with study authors to identify additional studies) in the search and date last searched.	5
Search strategy	8	Present full electronic search strategy for at least one database, including any limits used, such that it could be repeated.	5
Study selection	9	State the process for selecting studies (i.e., screening, eligibility, included in systematic review, and, if applicable, included in the meta-analysis).	5
Data collection process	10	Describe method of data extraction from reports (e.g., piloted forms, independently, in duplicate) and any processes for obtaining and confirming data from investigators.	5
Data items	11	List and define all variables for which data were sought (e.g., PICOS, funding sources) and any assumptions and simplifications made.	5
Risk of bias in individual studies	12	Describe methods used for assessing risk of bias of individual studies (including specification of whether this was done at the study or outcome level), and how this information is to be used in any data synthesis.	5
Summary measures	13	State the principal summary measures (e.g., risk ratio, difference in means).	Only in meta-analysis
Synthesis of results	14	Describe the methods of handling data and combining results of studies, if done, including measures of consistency (e.g., I2) for each meta-analysis.	Only in meta-analysis
Risk of bias across studies	15	Specify any assessment of risk of bias that may affect the cumulative evidence (e.g., publication bias, selective reporting within studies).	Only in meta-analysis
Additional analyses	16	Describe methods of additional analyses (e.g., sensitivity or subgroup analyses, meta-regression), if done, indicating which were pre-specified.	Only in meta-analysis

**RESULTS**			
Study selection	17	Give numbers of studies screened, assessed for eligibility, and included in the review, with reasons for exclusions at each stage, ideally with a flow diagram.	5,6
Study characteristics	18	For each study, present characteristics for which data were extracted (e.g., study size, PICOS, follow-up period) and provide the citations.	7–39
Risk of bias within studies	19	Present data on risk of bias of each study and, if available, any outcome level assessment (see item 12).	Table S5
Results of individual studies	20	For all outcomes considered (benefits or harms), present, for each study: (a) simple summary data for each intervention group (b) effect estimates and confidence intervals, ideally with a forest plot.	Only in meta-analysis
Synthesis of results	21	Present results of each meta-analysis done, including confidence intervals and measures of consistency.	Only in meta-analysis
Risk of bias across studies	22	Present results of any assessment of risk of bias across studies (see Item 15).	-
Additional analysis	23	Give results of additional analyses, if done (e.g., sensitivity or subgroup analyses, meta-regression [see Item 16]).	Only in meta-analysis
**DISCUSSION**			
Summary of evidence	24	Summarize the main findings including the strength of evidence for each main outcome; consider their relevance to key groups (e.g., healthcare providers, users, and policy makers).	39–46
Limitations	25	Discuss limitations at study and outcome level (e.g., risk of bias), and at review-level (e.g., incomplete retrieval of identified research, reporting bias).	39–46
Conclusions	26	Provide a general interpretation of the results in the context of other evidence, and implications for future research.	39–46
**FUNDING**			50
Funding	27	Describe sources of funding for the systematic review and other support (e.g., supply of data); role of funders for the systematic review.	

* From: Moher D, Liberati A, Tetzlaff J, Altman DG, The PRISMA Group (2009). Preferred Reporting Items for Systematic Reviews and Meta-Analyses: The PRISMA Statement. PLoS Med 6(7): e1000097. doi:10.1371/journal.pmed1000097.

Table S5. Checklist for assessing the quality of the studies.

Criteria		Yes (2)	Partial (1)	No (0)	N/A
1	Question/objective sufficiently described?				
2	Study design appropriate?				
3	Context for the study clear?				
4	Connection to a theoretical framework/wider body of knowledge?				
5	Characterization of the nanomaterial adequate?				
6	Cellular and Animal models of study appropriate?				
7	Use of In vitro and in vivo appropriate controls				
8	Analytical methods described/justified and appropriate?				
9	Results reported in sufficient detail?				
10	Conclusions supported by the results?				

[1] N/A was marked for items not applicable to a study design and were excluded from the calculation of the summary score. Summary scores of the studies were calculated based on the scoring of ten items by summing the total score obtained across the ten items and dividing by 20 (the total possible score).

Table S6. Biodistribution information and therapeutic outcomes obtained with rGO formulations.

Formulation	Therapeutic Strategy	Therapeutic Outcomes	Biodistribution	REF
rGONM-PEG-Cy7-RGD	- PTT (rGONM + Cy7)<br>- Target ligand RGD that directs formulation to integrin receptors overexpressed in cancer cells	Tumor ablated in 2 d<br>Survival ≥ 90 d	rGONM-PEG-Cy7 exhibited considerable tumor uptake, but also high uptakes in liver and spleen This indicates that non-targeted nanocarriers benefit from EPR effect and tend to locate at tumor sites and in RES organs.<br>RGD target ligand increases 3 × rGONM-PEG-Cy7-RGD uptake in comparison with rGONM-PEG-Cy7 and reduces organ uptake by 70%<br>48h after injection there is low uptake by organs indicating a fast clearance.	[17]
rGO nanosheets	- PTT (rGO + ICG)<br>- HA coating	Tumor ablated in 3 d	HArGO-ICG showed 47.7 × and 12.1 × higher distribution in tumor (immediately after injection or 48 h after injection) than at liver.	[18]
^{131}I-RGO-PEG	- PTT (rGO) + Radiotherapy (^{131}I)	Tumor ablated in 16 d	^{131}I-RGO-PEG had blood circulation half-lives of 0.65±0.27 h and 17.93±2.66 h.<br>Tumor accumulation (48 h after injection) was high (5% ID/g) but smaller than in liver (15% ID/g) and spleen (20% ID/g). This indicates that non-targeted nanocarriers benefit from EPR effect and tend to locate at tumor sites and in RES organs. In comparison free ^{131}I clearance is much faster which explains that tumor treated with ^{131}I is not ablated and volume increases 8 × in 18 d.	[19]
rGO-AuNRVe	- PTT (rGO) + Chemotherapy (DOX)	Tumor ablated in 14 d<br>Survival ≥ 40 d	rGO-AuNRVe tumor uptake increased from 0.79% ID/g (1 h after injection) to 3.68% ID/g (6 h after injection) to 9.7% ID/g (24 h after injection).<br>52 h after injection there was still ≈9.5% ID/g of rGO-AuNRVe in tumor.	[20]
FA-PEG-Lip@rGO/Res	- PTT (rGO) + Chemotherapy (Res)<br>- Target ligand FA that directs formulation to FA receptors overexpressed in cancer cells	Tumor ablated in 10 d<br>Survival ≥ 50 d	FA-PEG-Lip@rGO/Res were effectively uptaken by MCF-T breast cancer cells which are FA receptors (+) and were not uptaken by A549 normal lung cells which are FA receptors (-).	[21]
ArGO	- PTT (rGO)<br>- Bacteriomimetic poli-γ-glutamic surface coating	Tumor ablated in 10 d	ArGO was retained in tumor tissues at higher amounts than plain rGO.<br>ArGO tumor concentration (5 d after injection) was 2.1 × higher than plain rGO.<br>Greater distribution in tumor and prolonged tumor retention is explained by the bacteriomimetic surface modification.	[22]

Table S7. Biodistribution information and therapeutic outcomes obtained with GO, NGO and GQDs based formulations.

Formulation	Therapeutic strategy	Therapeutic outcomes	Biodistribution	REF
GO/MNWO$_4$/PEG	- PTT (GO+MnWO$_4$) + Chemotherapy (DOX)	Tumor ablated in 12 d and no recurrence observed until the end of study (16 d)	GO/MNWO$_4$/PEG accumulated in tumor and 24h after injection there was a tumor dose of Mn (7 % ID/g) indicating that there is still significant intratumor remaining of nanocarriers.	[23]
NGO-PEG-ICG/PTX	- Chemotherapy (PTX)	Tumor ablated in 15 d	NGO-PEG-ICG/PTX accumulated in tumor and 24h after injection there was a considerable tumor dose of ICG (29.1% ID/g) in comparison to ICG administrated alone (2.03% ID/g)<br>Accumulation is also observed in kidney and liver and may be attributed to the formulation metabolism and clearance.	[24]
CPGA	- PTT (GO+Cy5.5+Au) + PDT (Cy5.5)<br>- Target ligand MMP-14(P) that directs formulation to the overexpressed endoperoxidase in tumor cell membrane	Tumor ablated in 6 d and no recurrence observed until the end of study (14 d)	CPGA exhibited considerable tumor uptake (7.06% ID/g).<br>Accumulation is also observed in kidney and attributed to the formulation metabolism and clearance.	[25]
GO/AuNS-PEG/Ce6	- PTT (GO + AuNS) + PDT (Ce6)	Tumor ablated in 14 d and no recurrence observed until the end of study (21 d)	Mitochondria depolarization effects indicates that nanocarrier might escape from lysosomes to cytosol and target mitochondria, thus inducing cancer cell death.	[26]
NGO-IR-808	- PTT (NGO) + PDT (IR-808)<br>- Target ligand BPEI that directs formulation to cancer cells overexpressing organic anion transporting polypeptides (OATPs) receptors	Tumor ablated in 3 d	Mitochondria membrane potential was severely affected indicating ROS production and indicating that nanocarrier might escape from lysosomes to cytosol and target mitochondria, thus inducing cancer cell death.<br>Cellular uptake occurs by an energy dependent process through OATPS receptors.	[27]
GO-PEG-CysCOOH	- PTT (NGO+CysCOOH)	Tumor ablated in 2 d and no recurrence observed until the end of study (14 d)<br>Survival ≥ 60 d	GO-PEG-CysCOOH accumulates in tumor, liver and lung 24h after injection	[28]
GO/Bi_2Se_3/PVP	- PTT (GO+ Bi_2Se_3)	Tumor ablated in 2 d and no recurrence observed until the end of study (24 d)	GO/Bi_2Se_3/PVP is distributed in tumor, spleen, kidney, lung and liver 24h after injection<br>This indicates that non-targeted nanocarriers benefit from EPR effect and tend to locate at tumor sites and in RES organs. Kidney and liver accumulation can be also attributed to the formulation metabolism and clearance.	[29]
IR780/GQD-FA	- PTT (GQDs+ IR780)<br>- Target ligand FA that directs formulation to FA receptors overexpressed in cancer cells	Tumor ablated in 2 d<br>Survival ≥ 60 d	IR780/GQD-FA is distributed in tumor and less in liver and kidney while IR780 administrated alone occurs in liver, lung and kidney<br>Less distribution in liver and kidney indicates that targeting is effective and targeted nanocarriers tend to locate more at tumor sites instead of RES organs.	[30]

References

1. Ali, I.; Rahis, U.; Salim, K.; Rather, M.A.; Wani, W.A.; Haque, A. Advances in nano drugs for cancer chemotherapy. *Current cancer drug targets* **2011**, *11*, 135–146.
2. Amer, M.H. Gene therapy for cancer: Present status and future perspective. *Molecular and cellular therapies* **2014**, *2*, 27–27.
3. Zou, L.; Wang, H.; He, B.; Zeng, L.; Tan, T.; Cao, H.; He, X.; Zhang, Z.; Guo, S.; Li, Y. Current approaches of photothermal therapy in treating cancer metastasis with nanotherapeutics. *Theranostics* **2016**, *6*, 762–772.
4. Tabish, T.A.; Zhang, S.; Winyard, P.G. Developing the next generation of graphene-based platforms for cancer therapeutics: The potential role of reactive oxygen species. *Redox Biology* **2018**, *15*, 34–40.
5. Zhang, B.; Wang, Y.; Liu, J.; Zhai, G. Recent developments of phototherapy based on graphene family nanomaterials. *Current Medicinal Chemistry* **2017**, *24*, 268–291.
6. Spirou, S.V.; Basini, M.; Lascialfari, A.; Sangregorio, C.; Innocenti, C. Magnetic hyperthermia and radiation therapy: Radiobiological principles and current practice (†). *Nanomaterials (Basel, Switzerland)* **2018**, *8*, 401.
7. Xia, Y.; Matham, M.V.; Su, H.; Padmanabhan, P.; Gulyás, B. Nanoparticulate contrast agents for multimodality molecular imaging. *Journal of Biomedical Nanotechnology* **2016**, *12*, 1553–1584.
8. Smith, B.R.; Gambhir, S.S. Nanomaterials for in vivo imaging. *Chemical Reviews* **2017**, *117*, 901–986.
9. Lahooti, A.; Sarkar, S.; Laurent, S.; Shanehsazzadeh, S. Dual nano-sized contrast agents in pet/mri: A systematic review. *Contrast Media & Molecular Imaging* **2016**, *11*, 428–447.
10. Chen, J.; Zhao, J.X. Upconversion nanomaterials: Synthesis, mechanism, and applications in sensing. *Sensors (Basel, Switzerland)* **2012**, *12*, 2414–2435.
11. Wu, X.; Chen, G.; Shen, J.; Li, Z.; Zhang, Y.; Han, G. Upconversion nanoparticles: A versatile solution to multiscale biological imaging. *Bioconjugate Chemistry* **2015**, *26*, 166–175.
12. Kylili, A.; Fokaides, P.A.; Christou, P.; Kalogirou, S.A. Infrared thermography (irt) applications for building diagnostics: A review. *Applied Energy* **2014**, *134*, 531–549.
13. Kateb, B.; Yamamoto, V.; Yu, C.; Grundfest, W.; Gruen, J.P. Infrared thermal imaging: A review of the literature and case report. *NeuroImage* **2009**, *47*, T154-T162.
14. Cui, S.; Zhang, S.; Yue, S. Raman spectroscopy and imaging for cancer diagnosis. *Journal of Healthcare Engineering* **2018**, *2018*, 11.
15. Kong, K.; Kendall, C.; Stone, N.; Notingher, I. Raman spectroscopy for medical diagnostics — from in-vitro biofluid assays to in-vivo cancer detection. *Advanced Drug Delivery Reviews* **2015**, *89*, 121–134.
16. Zhang, Y.; Yu, J.; Kahkoska, A.R.; Gu, Z. Photoacoustic drug delivery. *Sensors* **2017**, *17*.
17. Akhavan, O.; Ghaderi, E. Graphene nanomesh promises extremely efficient in vivo photothermal therapy. *Small* **2013**, *9*, 3593–3601.
18. Miao, W.; Shim, G.; Kim, G.; Lee, S.; Lee, H.J.; Kim, Y.B.; Byun, Y.; Oh, Y.K. Image-guided synergistic photothermal therapy using photoresponsive imaging agent-loaded graphene-based nanosheets. *Journal of controlled release : official journal of the Controlled Release Society* **2015**, *211*, 28–36.
19. Chen, L.; Zhong, X.; Yi, X.; Huang, M.; Ning, P.; Liu, T.; Ge, C.; Chai, Z.; Liu, Z.; Yang, K. Radionuclide i-131 labeled reduced graphene oxide for nuclear imaging guided combined radio- and photothermal therapy of cancer. *Biomaterials* **2015**, *66*, 21–28.
20. Song, J.; Yang, X.; Jacobson, O.; Lin, L.; Huang, P.; Niu, G.; Ma, Q.; Chen, X. Sequential drug release and enhanced photothermal and photoacoustic effect of hybrid reduced graphene oxide-loaded ultrasmall gold nanorod vesicles for cancer therapy. *ACS Nano* **2015**, *9*, 9199–9209.
21. Hai, L.; He, D.; He, X.; Wang, K.; Yang, X.; Liu, J.; Cheng, H.; Huang, X.; Shangguan, J. Facile fabrication of a resveratrol loaded phospholipid@reduced graphene oxide nanoassembly for targeted and near-infrared laser-triggered chemo/photothermal synergistic therapy of cancer in vivo. *Journal of Materials Chemistry B* **2017**, *5*, 5783–5792.

22. Shim, G.; Kim, D.; Kim, J.; Suh, M.S.; Kim, Y.K.; Oh, Y.K. Bacteriomimetic poly-γ-glutamic acid surface coating for hemocompatibility and safety of nanomaterials. *Nanotoxicology* **2017**, *11*, 762–770.
23. Chang, X.; Zhang, Y.; Xu, P.; Zhang, M.; Wu, H.; Yang, S. Graphene oxide/mnwo4 nanocomposite for magnetic resonance/photoacoustic dual-model imaging and tumor photothermo-chemotherapy. *Carbon* **2018**, *138*, 397–409.
24. Zhang, C.; Lu, T.; Tao, J.; Wan, G.; Zhao, H. Co-delivery of paclitaxel and indocyanine green by pegylated graphene oxide: A potential integrated nanoplatform for tumor theranostics. *Rsc Advances* **2016**, *6*, 15460–15468.
25. Gao, S.; Zhang, L.; Wang, G.; Yang, K.; Chen, M.; Tian, R.; Ma, Q.; Zhu, L. Hybrid graphene/au activatable theranostic agent for multimodalities imaging guided enhanced photothermal therapy. *Biomaterials* **2016**, *79*, 36–45.
26. Wu, C.; Li, D.; Wang, L.; Guan, X.; Tian, Y.; Yang, H.; Li, S.; Liu, Y. Single wavelength light-mediated, synergistic bimodal cancer photoablation and amplified photothermal performance by graphene/gold nanostar/photosensitizer theranostics. *Acta Biomaterialia* **2017**, *53*, 631–642.
27. Luo, S.; Yang, Z.; Tan, X.; Wang, Y.; Zeng, Y.; Wang, Y.; Li, C.; Li, R.; Shi, C. Multifunctional photosensitizer grafted on polyethylene glycol and polyethylenimine dual-functionalized nanographene oxide for cancer-targeted near-infrared imaging and synergistic phototherapy. *ACS Applied Materials and Interfaces* **2016**, *8*, 17176–17186.
28. Rong, P.; Wu, J.; Liu, Z.; Ma, X.; Yu, L.; Zhou, K.; Zeng, W.; Wang, W. Fluorescence dye loaded nano-graphene for multimodal imaging guided photothermal therapy. *RSC Advances* **2016**, *6*, 1894–1901.
29. Zhang, Y.; Zhang, H.; Wang, Y.; Wu, H.; Zeng, B.; Zhang, Y.; Tian, Q.; Yang, S. Hydrophilic graphene oxide/bismuth selenide nanocomposites for ct imaging, photoacoustic imaging, and photothermal therapy. *Journal of Materials Chemistry B* **2017**, *5*, 1846–1855.
30. Li, S.; Zhou, S.; Li, Y.; Li, X.; Zhu, J.; Fan, L.; Yang, S. Exceptionally high payload of the ir780 iodide on folic acid-functionalized graphene quantum dots for targeted photothermal therapy. *ACS Applied Materials and Interfaces* **2017**, *9*, 22332–22341.
